# Lignin Structure and Reactivity in the Organosolv Process Studied by NMR Spectroscopy, Mass Spectrometry, and Density Functional Theory

**DOI:** 10.1021/acs.biomac.3c00186

**Published:** 2023-04-20

**Authors:** Maria Karlsson, Joakim Romson, Thomas Elder, Åsa Emmer, Martin Lawoko

**Affiliations:** † Wallenberg Wood Science Center, Department of Fiber and Polymer Technology, School of Chemistry, Biotechnology and Health, KTH Royal Institute of Technology, Teknikringen 56-58, SE-100 44 Stockholm, Sweden; ‡ Analytical Chemistry, Division of Applied Physical Chemistry, Department of Chemistry, School of Engineering Sciences in Chemistry, Biotechnology and Health, Royal Institute of Technology, KTH Teknikringen 36, SE-100 44 Stockholm, Sweden; § USDA-Forest Service, Southern Research Station, 521 Devall Drive, Auburn, Alabama 36849, United States; ∥ Division of Wood Chemistry and Pulp Technology, Department of Fiber and Polymer Technology, School of Chemistry, Biotechnology and Health, KTH Royal Institute of Technology, Teknikringen 56-58, SE-100 44 Stockholm, Sweden

## Abstract

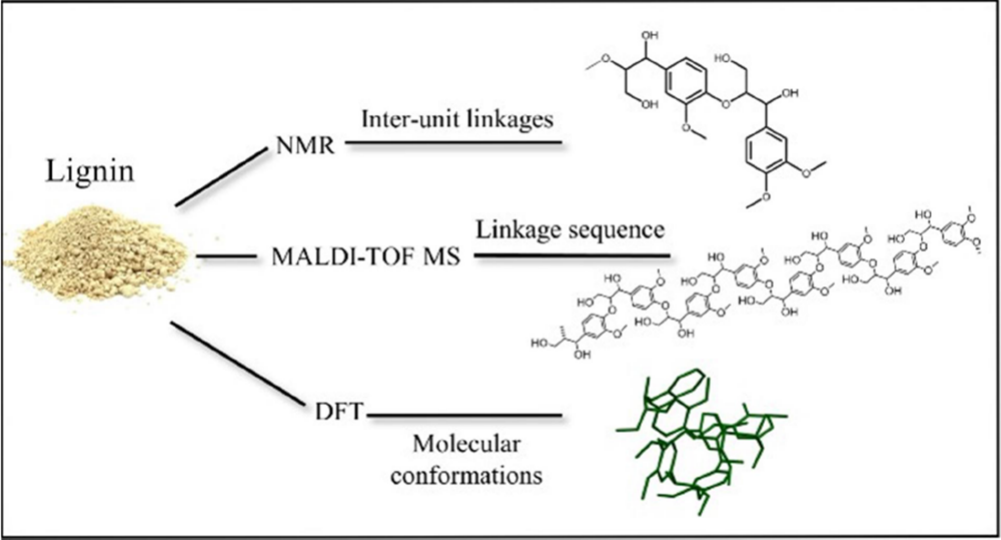

There is need for well-defined lignin macromolecules for research related to their use in biomaterial and biochemical applications. Lignin biorefining efforts are therefore under investigation to meet these needs. The detailed knowledge of the molecular structure of the native lignin and of the biorefinery lignins is essential for understanding the extraction mechanisms as well as chemical properties of the molecules. The objective of this work was to study the reactivity of lignin during a cyclic organosolv extraction process adopting physical protection strategies. As references, synthetic lignins obtained by mimicking the chemistry of lignin polymerization were used. State-of-the-art nuclear magnetic resonance (NMR) analysis, a powerful tool for the elucidation of lignin inter-unit linkages and functionalities, is complemented with matrix-assisted laser desorption/ionization-time-of-flight-mass spectrometry (MALDI-TOF MS), to gain insights into linkage sequences and structural populations. The study unraveled interesting fundamental aspects on lignin polymerization processes, such as identifications of molecular populations with high degrees of structural homogeneity and the emergence of branching points in lignin structure. Furthermore, a previously proposed intramolecular condensation reaction is substantiated and new insights into the selectivity of this reaction are introduced and supported by density functional theory (DFT) calculations, where the important role of intramolecular π–π stacking is emphasized. The combined NMR and MALDI-TOF MS analytical approach, together with computational modeling, is important for deeper fundamental lignin studies and will be further exploited.

## Introduction

Lignin constitutes about 15–30% of lignocellulosic biomass and is the most abundant natural source of aromatics on earth with the potential to replace fossil-based phenols in material applications.^1^ However, due to the heterogeneity of lignin, its chemical and physical properties still require deeper investigations. The increased fundamental knowledge about lignin properties is of relevance for understanding the reactivity of lignin under certain process conditions, predicting reactions and subsequently developing valorization strategies for extracted lignin.^2^

Modern lignin biorefining concepts adopt chemical and physical protection strategies to prevent intermolecular condensation reactions.^3^ The protected lignin with high amounts of aryl ether linkages (β-O-4′) is especially suitable for catalytic depolymerization to small oligomers and monomers, which is interesting for further use as platform chemicals and fuel.^4,5^ In chemical protection, additives are applied to endcap reactive sites on the lignin molecule, such as carbocations formed on the aliphatic sidechain. The use of aldehydes as additives (aldehyde-assisted fractionation, AAF) is one example.^6−8^ Physical protection, where the lignin structure is protected by the design of the extraction setup, has been achieved by use of the flow-through extraction concept where a continuous extraction is performed, yielding a lignin with an abundance of native linkages.^9^

Nuclear magnetic resonance (NMR) techniques constitute the state-of-the-art for the structural analysis of lignin, where 1D and 2D NMR methods have been very useful for analysis of lignin inter-unit linkages and functionality in milled wood lignins^10−12^ and technical lignins.^13−15^ The most common NMR techniques are heteronuclear single quantum coherence (HSQC) NMR for lignin inter-unit linkages, ^31^P NMR to quantify the hydroxyl functionalities, and quantitative ^13^C NMR.

However, in NMR, the bulk of the lignin is characterized, and there are no differentiations between populations. This problem is partly resolved by combining a separation step followed by mass spectrometry (MS) detection, where various ionization sources, with or without tandem MS (MS/MS), have been evaluated.^16−20^ The analysis and fragmentation of model compounds are useful to obtain knowledge of the fragmentation pattern of lignin itself, from which tentative structures of molecular lignin can be obtained.^21^ Non-target screening and multiple-stage tandem MS in combination with classification models have been used to identify related lignin structures.^18,22^

Matrix-assisted laser desorption/ionization (MALDI) is a soft ionization technique that can potentially ionize lignin oligomers intact.^23^ To separate the generated ions, the mass analyzer time-of-flight (TOF) is often used, where the ions are separated according to the mass-to-charge ratio reflected in their flight time in the tube.^24^ The TOF MS can either be used in linear or reflectron mode. The reflectron technology is more useful for improved resolution.^25^

The sample preparation has an impact on the outcome of the MS analysis, where MALDI has the advantage that the analyte does not have to be soluble in the matrix mixture.^26^ The complexity of lignin in terms of structure, solubility, and dispersity positions MALDI-TOF MS as a potentially useful technique for its analysis. However, there are limitations, such as low ionization efficiency which can be traced back to the structural and chemical features of lignin and also causes challenges in forming homogeneous matrix mixtures.^27^ The matrix selection possibly affects what part of the lignin can be detected.^28^ Attempts to increase the desorption and ionization efficiency of lignin have been made, by using, for example, novel matrices based on ionic liquids.^27^

Among others, a commonly used matrix for lignin is 2,5-dihydroxybenzoic acid (2,5-DHB).^29^ Lignin is often analyzed in both positive and negative modes. In one study, the ionization efficiency for low molecular weight lignin was shown to be better in positive mode while high molecular weight lignin was ionized more efficiently in negative mode when using the matrices α-cyano-4-hydroxycinnamic acid/α-cyclodextrin. This combination also suppressed the signal related to the matrix.^30^ To assess lignin polymerization, MALDI-TOF MS studies have been performed on dehydrogenation polymers (DHP, synthetic lignin).^31,32^

Another analysis used in the present study is density functional theory (DFT). DFT calculations have been extensively applied to lignin model compounds. The literature includes large studies of the reactivity of dimeric models,^33,34^ examination of reactions occurring under pyrolysis conditions,^35−39^ interpretation of Raman spectroscopy,^40^ and the incorporation of non-canonical monomers into the lignin polymer.^41−44^ Work by Houston et al.^37^ and Azad et al.^38,39^ is notable for the size of the models that were examined, tetramers and decamers, respectively. Over the recent past, the M06-2X^45^ method has been predominately used in this type of work.

In our earlier work,^2,46^ we developed an ethanol-based organosolv lignin biorefinery process using an additive-free physical protection concept, where the native lignin structure is preserved to a high degree. The physical protection is attributed to the cyclic extraction methodology.

In the present study, we combine different NMR methodologies, MALDI-TOF MS, and DFT calculations to gain deeper insights into lignin structure and the reactivity during the ethanol-based organosolv performed in cyclic mode. Synthetic lignins prepared by mimicking lignin polymerization chemistry are used as references for comparison in the MALDI-TOF MS analysis.

## Materials and Methods

### Chemicals

The lignin sample was extracted from Norway spruce (*Picea abies*). Sand (50–70 mesh), iron(III) chloride hexahydrate (reagent grade), coniferyl aldehyde (98%), ethyl acetate (anhydrous, 99.8%), sodium ascorbate (≥98%), pyridine (anhydrous, 99.8%), *endo-N*-hydroxy-5-norbornene-2,3-dicarboximide (*e*HNDI; 97%), chromium(III) acetylacetonate (Cr(acac3); 99.99%), 2-chloro-4,4,5,5-tetramethyl-1,3,2-dioxaphospholane (Cl-TMDP; 95%), [D6]DMSO (99.9% D), *N,N*-dimethylformamide (anhydrous, 99.8%), CDCl3 (≥99.8 atom % D), trifluoroacetic acid (99%), magnesium sulfate (≥97%), DMSO (99.7%), and toluene (≥99.5%) were all purchased from Sigma-Aldrich. 2,5-DHB was purchased from Bruker (Bruker Daltonik). Silica gel (ultra-pure, 40–60 μm, 60A) and sodium borohydride (NaBH4, 99%) were purchased from Acros Organics. Heptane (≥99%), acetone (≥99%), methanol (≥99.8%), ethanol (99.8%), and tetrahydrofuran (HPLC grade) were purchased from VWR. Sodium chloride (reagent grade) was purchased from Scharlau. Sulfuric acid (>95%, analytical grade), acetic anhydride (99.7%, analytical grade), and acetonitrile (≥99.8%, HPLC grade) were purchased from Fischer Chemicals. All water used in the experiments refers to Milli-Q water (Millipore, Q-POD, Millipak 0.22 μm filter, 18.2 MΩcm).

### Wood Preparation and Extraction Equipment

Wood chips from spruces were milled to a size of 40 mesh using a Wiley mini mill (3383 L70, Thomas Scientific).

The extractions were performed using an accelerated solvent extractor, ASE 350 (Dionex, Sunnyvale, CA, USA) instrument, together with 66 mL dionium zirconium extraction cells (Dionex, Sunnyvale, CA, USA) using glass fiber filters.

### Separation of Dimers

Column chromatography was performed using a slurry-packed column with silica gel (40–60 μm). Thin layer chromatography was made using an aluminum plate coated with silica (TLC Silica gel 60 F254, Merck Millipore). The dimers were detected using UV light (254 nm).

### Size Exclusion Chromatography (SEC)

The setup of the SEC system consisted of an autosampler (2707), HPLC pump (515), and a photodiode array detector (2998) operated at 254 and 280 nm (Waters Milford, MA, USA). The following column system was used for the separation: Waters Styragel Guard column (4.6 × 300 mm) in series with Waters Ultrastyragel HR4, HR2, and HR0.5 (4.6 × 300 mm). The calibration was performed using polystyrene standards at 254 nm, with the nominal molecular weights ranging from 162 to 176,000 Da (specifically, 176,000, 116,000, 46,400, 18,000, 9600, 6540, 2920, 890, 578, 474, 370, 266, and 162 Da). Data acquisition and processing were performed using the Waters Empower 3 build 3471 software.

### NMR Spectroscopy

The NMR spectra were acquired using the Bruker NMR spectrometer Avance III HD 400 MHz (Bruker Corporation, Billerica, MA, USA) instrument. The instrument was equipped with a 5 mm Z-gradient BBFO broadband smart probe (Bruker Corporation, Billerica, MA, USA). The data processing was performed in MestReNova (v.9.0.0, Mestrelab Research).

### Matrix-Assisted Laser Desorption/Ionization Time-of-Flight Mass Spectrometry (MALDI-TOF MS)

The MALDI-TOF MS was performed on a Bruker ultrafleXtreme (Bruker Daltonik, Leipzig, Germany) instrument equipped with a Nd:YAG laser (355 nm) and a reflectron. The data acquisition and processing were performed using flexControl 3.4 and flexAnalysis 3.4 (Bruker Daltonik).

### Lignin Extraction from Wood

The extraction method was based on a previously published method.^46^ To minimize systematic errors, all extractions were strictly following a protocol. The wood chips were ocularly inspected, and wood with visible deviations was not included in the experiments. The wood chips were milled to 40 mesh using a Wiley mill.

For all experiments, 9.281 g of oven-dry-based Wiley milled wood was placed in a 66 mL dionium zirconium extraction cell. In the first step, a hot water extraction was performed, with an extraction time of 2 h. The program parameters were as follows: a fixed volume of 70 mL, 160 °C, and a purge time of 90 min. The fixed volume was set to reach the pressure of 1500–1600 psi.

The system was rinsed with the new solvent system, and subsequently, the organosolv extraction of lignin immediately started. The binary solvent system of ethanol/water was used with sulfuric acid as a catalyst. The extraction was performed in 15 static cycles for 5 min, using the standard method. The program parameters were as follows: rinse volume of 100% and a purge time of 90 s.

The lignin sample was refined by ethanol fractionation, where lignin and ethanol were mixed (1:40 w/w, lignin/ethanol) in a closed vial for 2 h under stirring. The solution was filtered under vacuum, and the ethanol was removed from the soluble lignin filtrate under reduced pressure by rotary evaporation.

### Sample Preparations for Analysis

The dispersity, *Đ*, was determined using a THF-based system. To increase the solubility of the lignin, acetylation was performed prior to analysis.

Acetylation was performed according to Gellerstedt;^47^ briefly, 2.5 mg of lignin was dissolved in 100 μL of acetic anhydride and 100 μL of pyridine. The mixture was stirred overnight at ambient temperature using a thermomixer at 400 rpm. The solvents were evaporated using a stream of nitrogen, and simultaneously, a few drops of the mixture of toluene/methanol (1:1) were added until dryness. The residue was dissolved in 1 mL THF. Prior to analysis, the dissolved lignin was filtered using a 0.2 μm NYL syringe filter. The chromatography was performed using THF as eluent at a flow rate of 0.3 mL/min, injection volume of 20 μL, and a column temperature of 35 °C.

For the HSQC NMR analysis, 80 mg of extracted lignin and 30 mg of the lignin references, respectively, were dissolved in 600 μL of DMSO-d6. The spectra were acquired using the pulse program “hsqcetgpsi”, with a relaxation delay of 1.5 s, 86 scans, and an acquisition time of 0.11 s at a temperature of 297 K, using 1024 × 256 increments. The data were processed using a 90°-shifted square sine-bell apodization window and 1024 × 1024 data points. The data were Fourier transformed and baseline corrected by a Bernstein polynomial fit of the third order in the ^1^H and ^13^C dimensions.

The sample for ^31^P NMR analysis was prepared according to previously reported method;^48,49^ briefly: 30 mg of lignin was dissolved in equal volumes of *N,N*-dimethylformamide (100 μL) and pyridine (100 μL), followed by the addition of 50 μL of an internal standard solution (60 mg ml^–1^ eHNDI in pyridine, 5 mg ml – 1 Cr(AcAc3) relaxing agent). Phosphorylation was performed by adding 100 μL of the phosphorylating agent (Cl-TMDP). Finally, 450 μL of CDCl3 was dropwise added to the solution.

The lignin samples for MALDI-TOF-MS analysis were prepared using DMSO as a diluent for a concentration of 0.05 mg/mL. The matrix was prepared by 10 mg/mL 2,5-DHB in TA30 (30:70 (v/v) acetonitrile/0.1% trifluoroacetic acid in water). Finally, 10:1 (v/v) ratio of matrix solution/lignin sample solution was mixed and 0.5 μL of droplets were applied on the MALDI plate. The mass spectrum was generated by 4000 laser shots. A peptide calibration standard (Bruker peptide calibration standard II) was used as an external calibration standard in positive mode.

MALDI MS analysis using the CHCA, 2,5-DHB, and DHAP as matrices and without matrix was investigated. Different sample diluents were investigated, more specifically, DMSO and THF as single diluents. Additionally, a mixture of the binary solvent systems of ethanol/water, ethanol/THF, and the tertiary mixture of ethanol/water/THF was also investigated as diluents. Different analyte concentrations were tried in combination with different matrix solvents, such as acetonitrile ethanol, THF, and the mixture TA30. Additives such as pyridine and TFA were investigated. Finally, the matrix-to-sample ratio and droplet size were evaluated. Both positive and negative modes were evaluated. Different methods of mixing the matrix and analyte were investigated: pre-mixing of the matrix and sample, and s-m-s and m-s-m layer formation, where the lignin sample (s) and the matrix (m) were added as separately dried layers onto the target.

### Synthesis of Lignin Dimers

#### Step 1: Reduction of Coniferyl Aldehyde to Coniferyl Alcohol

First, coniferyl aldehyde was reduced to coniferyl alcohol. The method was based on previously reported methods.^50−52^ Shortly, 0.4267 g of NaBH4 was added to 60 mL of ethyl acetate in a round bottom flask under stirring. To the mixture, 1 g of coniferyl aldehyde was added. The mixture was stirred for 17.5 h at room temperature. Approximately 67 mL of water was added to quench the reaction, followed by additional stirring for 30 min.

The mixture was placed in a separatory funnel, and the aqueous phase was extracted with ethyl acetate several times to ensure that most products were extracted from the aqueous phase. The pooled organic phases were washed with brine, and the aqueous phase was separated. The organic phase was thereafter dried with MgSO4. The solution was filtered using a glass fiber filter, and thereafter, the ethyl acetate was evaporated at low pressure using a rotary evaporator. The oily product, still containing ethyl acetate, was further dried at low pressure. The purity of the light-beige crystallized product was investigated by ^1^H NMR, Figure S7, to examine if further purification was needed. From the ^1^H NMR spectra, no traces of coniferyl aldehyde were visible and the product was determined to be used directly for dimer production. The yield of the coniferyl alcohol was 73.5%.

#### Step 2: Synthesis of Lignin Dimers β-O-4′, β-5′, and β-β′

This method follows the previously published method.^53^ The coniferyl alcohol from step 1, 0.7347 g, was dissolved in approximately 21 mL of acetone and diluted with 0.205 dm^3^ of water. To the mixture, a solution of 1.212 g of iron(III) chloride hexahydrate in 4.6 mL of water was added and stirred for 1 hour and, after that, extracted with ethyl acetate 6 × 30 mL using a separatory funnel. The pooled extract was washed with 31 mL of 0.1 M sodium ascorbate and 61 mL of brine, and dried with MgSO4. The solution was filtered using a glass fiber filter, and thereafter, the ethyl acetate was evaporated at low pressure. The oily product, still containing ethyl acetate, was further evaporated using rubber and septum. The mixture of dimers was finally dried under a vacuum overnight at room temperature.

#### Step 3: Separation of Dimers

Briefly, different solvent systems were tested with TLC to determine a solvent system to separate the dimers. Since the dimers are good absorbers of UV light themselves, no staining was necessary to visualize them under UV light. The mixture of dimers was separated by flash column chromatography. The column was slurry-packed using approximately 100:1 silica/sample ratio. Since the column had a built-in glass filter, sand only needed to be added on top.

The dimer mixture was dissolved in acetone. The final solvent system selected for the separation was 50% acetone/50% heptane. Every second fraction (of approximately 30 mL) was investigated by TLC, and when all three dimers had eluted, the mobile phase was changed to 80% acetone and later 100% acetone. The fractions were divided according to purity, and the separated dimers were evaporated at low pressure using a rotary evaporator and further dried under vacuum at room temperature over-night. The dimers were finally identified by HSQC NMR and MALDI-TOF MS analysis.

### Density Functional Theory Calculations

Computational chemistry methods were applied to the structures shown in Figure 10. These hexamers have multiple rotatable bonds and therefore can be quite flexible. As such, a conformational search is critical for identifying the more stable conformations that would be more probable in a population. A 1000 step Monte Carlo search was performed with the MMFF force field minimization. A maximum of 500 conformers within a 40 kJ mol^–1^ window of the minimum were subsequently refined by optimization using the PM6 semi-empirical method. These steps were executed using Spartan’203. The 10 lowest energy conformations were subsequently optimized using the M06-2X density functional method, the 6-31 + G(d) basis set, GD3 empirical dispersion correction, and DMSO solvation with the SMD model as implemented in Gaussian 164. The measurements in Figures 10 and 11 were made on lowest energy conformer from the density functional theory optimizations using Mercury 20225.

## Results and Discussion

### Purification of Lignin References

Lignin references were synthesized by radical coupling reactions and subjected to the same analytical methodologies. The synthesis was based on the protocols,^50−52^ starting with the reduction of coniferyl aldehyde to coniferyl alcohol followed by the synthesis of lignin dimers^53^ and, in the end, separation by column chromatography. The dimer references were well separated by column chromatography and eluted in the order of β-β′, β-5′, and β-O-4′. Two oligomeric fractions were also isolated with column chromatography. These references serve a dual purpose: (1) as qualitative standards for the MALDI-TOF MS analysis of lignin and (2) as references for native lignin structure to assess changes during organosolv extraction, and associated mechanisms.

### Molar Mass of Protected Lignin by SEC

We have recently developed a lignin biorefinery process that adopts a physical protection strategy yielding a lignin product with preserved native linkages.^2,46^ The organosolv process applies ethanol/water (70:30,wt/wt) and 1.5 wt % sulfuric acid, extracting in cycles at 160 °C. Analysis of the crude lignin product by SEC showed a broad dispersity (*Đ*) (Table S1, with a *Đ* value of 3.9); hence, a refinement to obtain low dispersity materials was performed using pure ethanol as solvent at ambient conditions to yield ethanol soluble and insoluble fractions, both with low *Đ* values (1.8 and 3.0, respectively, Tables 1 and S1). The fractionation method was performed according to previous reports.^46^

**Table 1:** *M*_n_, DP*n*, and *Đ* of the Lignin and Synthesized Oligomeric Fraction Using THF-SEC (Table S1), *n* = 2^a^

sample	*M*_n_	*M*_w_	DP_*n*_	*Đ*
ethanol soluble cyclic extracted lignin	1100 ± 30	1900 ± 57	6	1.8 ± 0.1
synthesized oligomeric lignin	600 ± 16	650 ± 19	3	1.1 ± 0.010

a The DP*n* calculation is based on a general mass of 180 Da, representing a monomeric repeating unit based on coniferyl alcohol, and is often used to determine the degree of polymerization.

The ethanol soluble fraction (*Đ* of 1.8) will be the focus of this study. The fraction accounts for approximately half (46%) of the total mass of the crude lignin sample. The degree of polymerization (DP*n*) of this fraction based on the number average molar mass was estimated to about 6, Table 1.

### Matrix Screening for the MALDI-TOF MS Analysis of Lignin

Screening of different matrices was performed and compared to references where no matrix was used, with the protected lignin obtained from the biorefinery process as substrate. The combinations giving the highest S/N ratio for the signals present in the lignin regions were all recorded in positive mode, using DMSO or EtOH/H_2_O (70: 30 v/v) as diluent with 2,5-DHB in TA30 as a matrix. The spectra presented in this study (Figures 3 and 7 and S1–S6) are recorded using DMSO as diluent. The methods were evaluated based on the number of peaks in the oligomeric region of the spectrum.

### Analysis of Dimeric Lignin References

The presence of dimers in the three fractions was investigated using MALDI-TOF MS and HSQC NMR. It was clear from both analyses that the chromatographic column separation method exhibited high selectivity in the separation of the dimers (Figures S8–S10 and S1–S3). More specifically, the HSQC NMR analysis confirmed that separation was inter-unit specific yielding the β-β′, β-5′, and β-O-4′ in separate fractions, while MALDI-TOF MS confirmed the mass of the corresponding sodium and potassium adduct ions of the dimers. The MALDI-TOF MS spectra (Figures S1–S3) recorded in positive ion mode show dominance of the sodium adduct cation for the three dimers, specifically, the monoisotopic masses of (β-O-4′): *m/z* 399.35, (β-5′): *m/z* 381.37, and (β-β′): *m/z* 381.37 (Structures **1**, **2**, and **3**, respectively, Figure 4). The presence of both the sodium and the potassium cation was used as an indication of the presence of the dimer reference. The formation mechanism of these dimers is shown in Figure S12. Similar mechanistic principles apply for the formation of oligomers discussed in the next section.

### Analysis of Oligomeric Fractions

**Figure 1:**
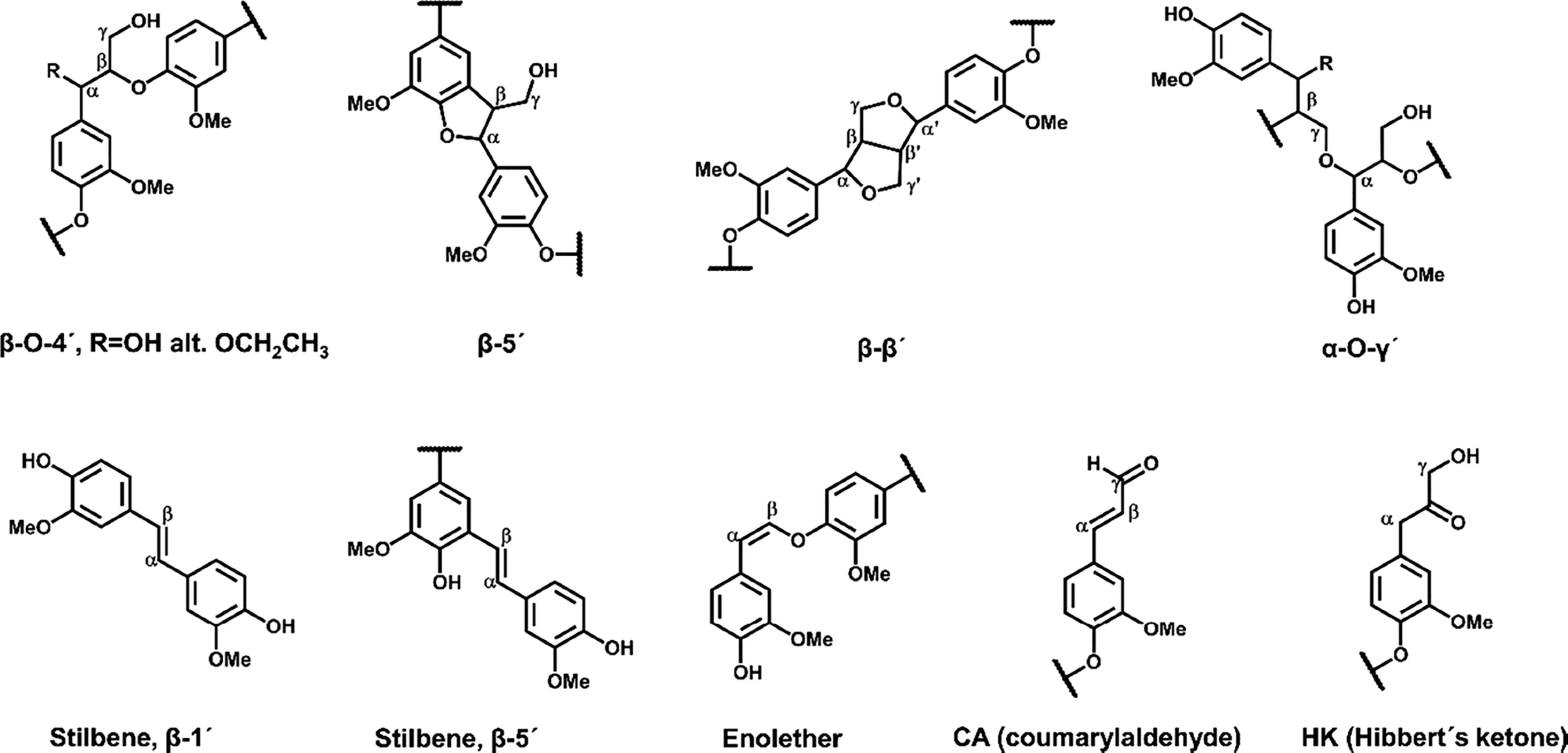
Lignin inter-unit linkages including both native structures and structures formed under organosolv extraction conditions.

**Figure 2:**
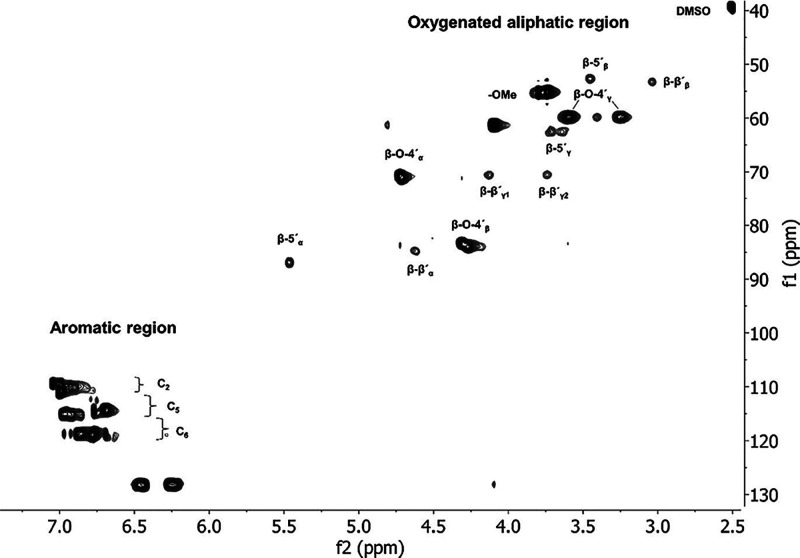
HSQC NMR spectra of oligomeric synthesized reference.

**Figure 3:**
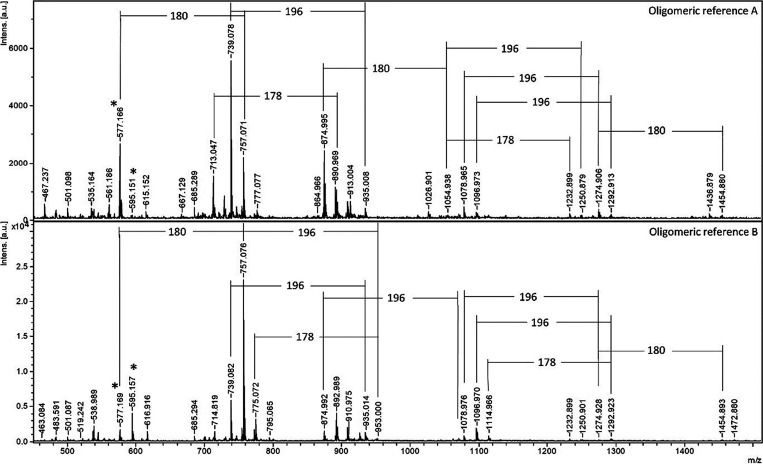
MALDI-TOF MS spectra of lignin oligomeric references A and B. The mass increments of 178, 180, and 196 are indicated, together with an asterisk (*) for the mass increments of 196 and 178 of the dimeric β-O-4′, β-5′, and/or β-β′, Figures S1–S3.

The oligomeric fraction of the synthesized references was characterized by HSQC NMR for inter-unit linkages, Figures 1 and 2, and MALDI-TOF MS for cluster analyses and related mass, Figure 3. HSQC analyses show the dominance of β-O-4′ inter-unit linkages and only small amounts of β-5′ and β-β′, clearly in line with what has been proposed for native lignin.^54^

The trimeric to the octameric region of the MALDI spectra of the oligomeric references are reported in Figures 3 and S4, respectively, where the matrix spectrum is included for both of the oligomeric references. Uniformly distanced clusters can be observed in the studied region for both the references A and B, Figure 3. The cluster formation shows a mix of the oligomeric fractions with increasing mass in the MALDI MS analysis. The highest mass observed in MALDI MS was 1472 Da, which corresponds to approximately eight aromatic monomers.

#### Trimeric Structures

**Figure 4:**
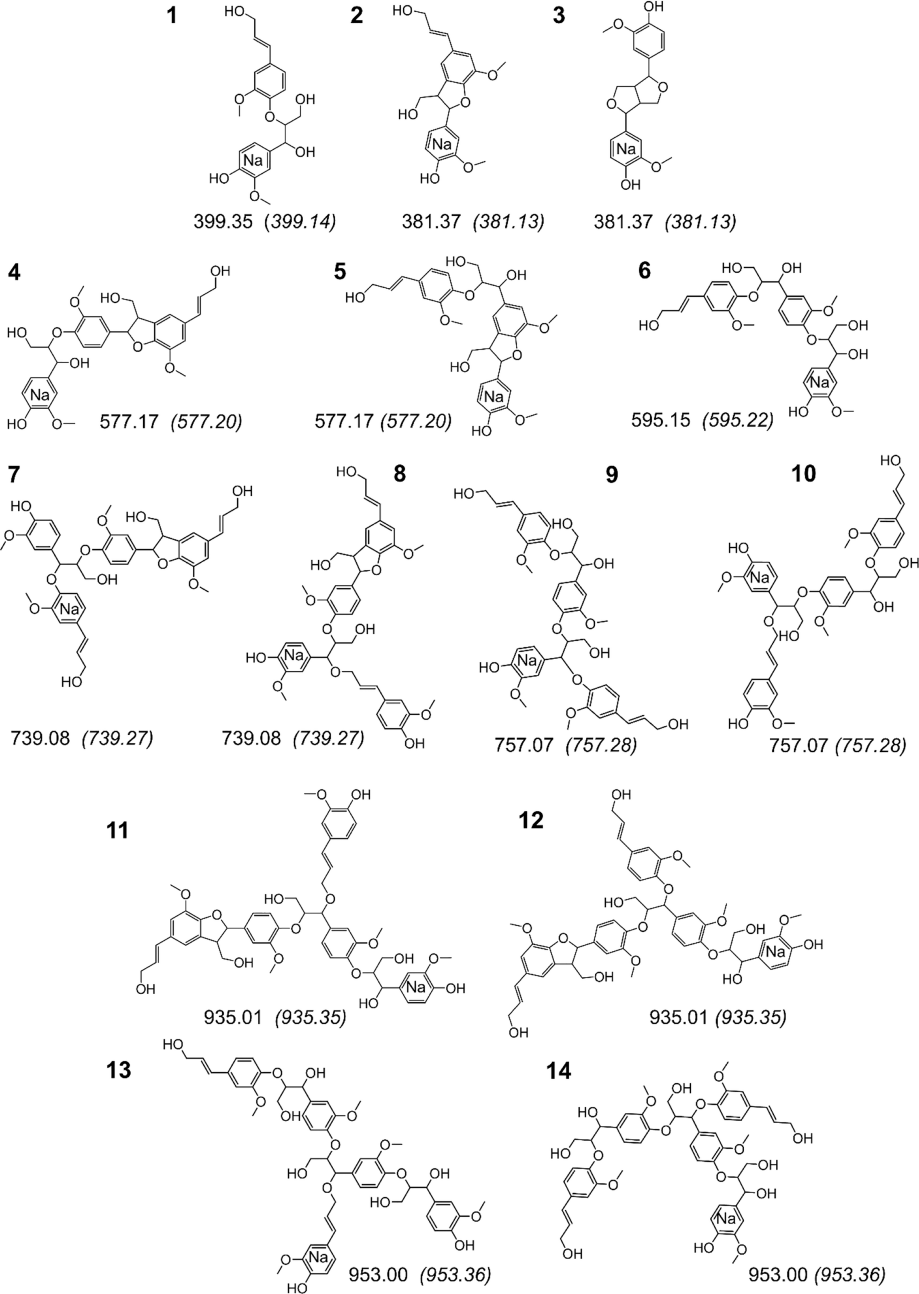
Tentative structures derived from MALDI-TOF MS cluster analyses. The *m/z* ratios of the measured monoisotopic mass are inserted beside each structure together with the calculated monoisotopic *m/z* values shown in respective parentheses.

An asterisk indicates the identified lignin trimers which are built from the aforementioned dimers, Figures S1–S3. The signal *m/z* 577.17 is assigned to two trimers; Structures **4** and **5**, Figure 4. Structure **4** results from a mass increment of 178 starting from the sodium adduct of dimeric β-O-4′ with the *m/z* 399.35 (Structure **1**, Figure 4). It is formed by the radical coupling of a β radical to a radical on the 5′ position on the next ring, followed by internal trapping reactions to yield β-5′ phenylcoumaran sub-structure. This follows the coupling principles shown in Figure S12,C. Structure **5** results from a mass increment of 196 starting from a preformed dimeric β-5′ (Structure **2**, *m/z* 381.37, Figure 4). The mass increment of 196 is a signature for the formation of a β-O-4′ linkage through radical coupling and subsequent addition of water to the formed quinone methide intermediate, following the coupling principles in Figure S12,B. Structure **6**, *m/z* 595.16, is a β-O-4′ trimer originating from the dimeric β-O-4′ (Structure **1***m/z* 399.35) by an endwise addition.

#### Tetramers

**Figure 5:**
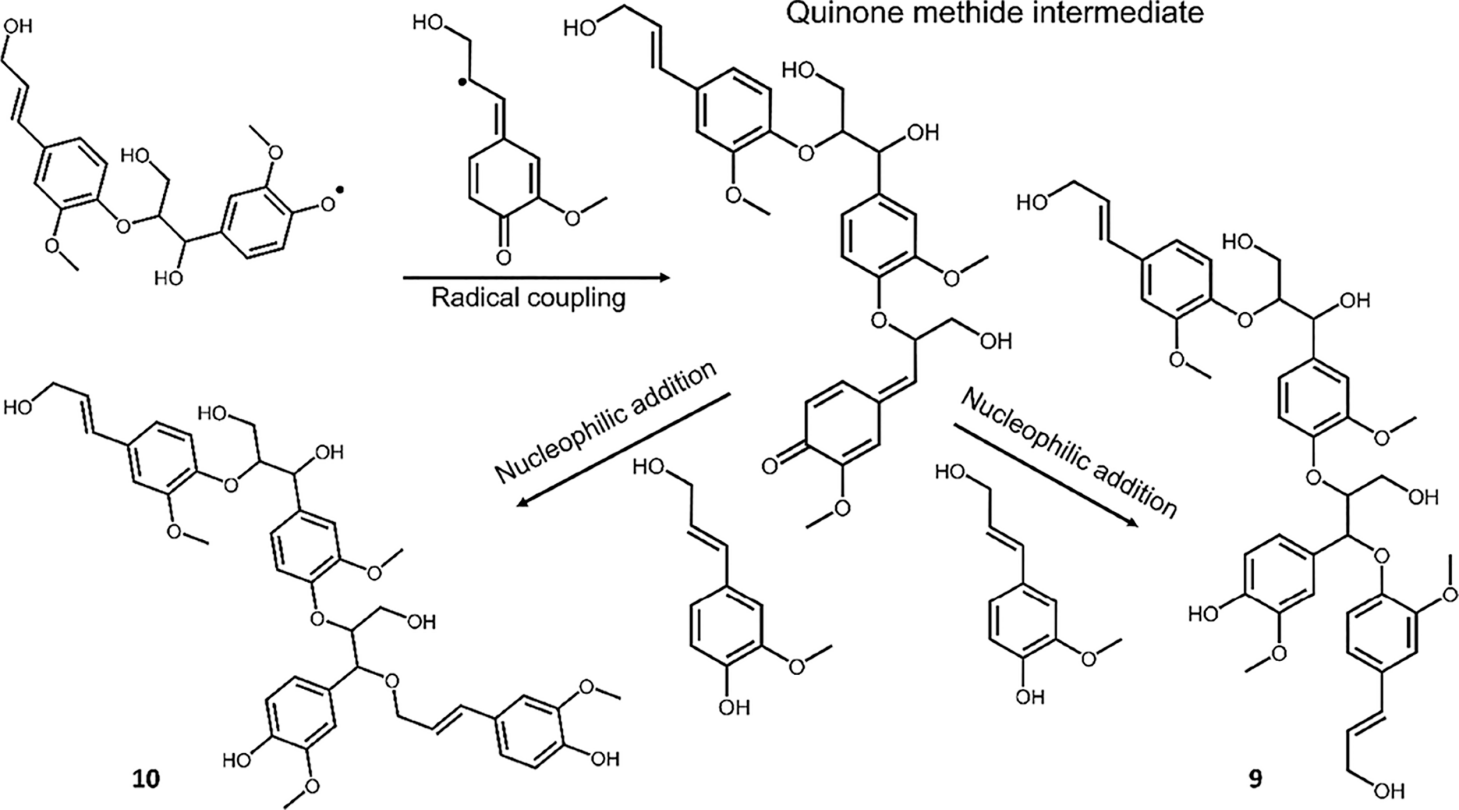
Emergence of branching points in bio-mimicked lignin tetramers.

Four candidates are identified (Structures **7**, **8**, **9**, and **10**). Interestingly, these tetramers could be formed by consecutive addition of coniferyl alcohol in different forms, to preformed dimers (Structures **1** and **2**), and by different mechanisms (Figure 5). The first mechanism involves radical coupling to yield a trimeric quinone methide intermediate and results in a mass increment of 178. The second mechanism, occurring subsequent to the first, is a nucleophilic addition of hydroxyls in coniferyl alcohol to the quinone methide, which yields an additional mass increment of 180. The proposed reaction pathway of the described reactions yielding **9** and **10** is shown in Figure 5. The benzylic ethers formed are interesting since they constitute branching points in lignin. It is noteworthy that these benzylic ethers were not detected by HSQC NMR in the oligomers (Figure 2) implying that they are present in small amounts. This is not surprising as the addition of water to form the benzylic alcohol dominates and is the main product from the HSQC studies. Nevertheless, the detection of branching points by MALDI-TOF MS supports the recent NMR studies on milled wood lignin.^10,12^ Similar reaction pathways to that shown in Figure 5 yields **7** and **8**.

#### Pentamers

Structures **11** and **12** (Figure 4) are pentamers formed from Structures **7** and **8** with a mass increment of 196, i.e., formation of a new β-O-4′ linkage. Structures **13** and **14** are formed similarly from Structures **9** and **10**.

Higher masses were also detected, but these were more difficult to decipher. Nevertheless, in the regions between *m/z* 1000 and 1500, mass increments of 196 (signature for β-O-4′ formation), 178 (β-5′ and β-β′), and 180 which as discussed are a signature for nucleophilic addition of coniferyl alcohol to a quinone methide intermediate. All these increments are consistent with what was discussed for the smaller oligomers and hence lend credence to the observation that such larger molecules may constitute lignin structures, and that the endwise polymerization may be preferred over bulk polymerization.^55^

To summarize this part, analysis of the lignin references using the combined NMR and MALDI-TOF MS approaches provides fundamental insights on the lignin polymerization and structural populations. The MALDI analysis of the synthesized lignins showed that branched oligomers could be formed during lignin polymerization. As a word of caution, the plant cell wall conditions cannot be accurately reproduced by classical lignin polymerization biomimicry approaches. The relative inter-unit abundancies are affected by the exact polymerization environment. However, the high content of β-O-4′ inter-units in the studied oligomers in this work is consistent with the recent literature on native lignin structure.^54^ This lends credence to the biomimicry attempts as being fairly representative in terms of the production of native-like lignin.

### Analysis of Spruce Organosolv Lignin from the Cyclic Extraction Process

**Figure 6:**
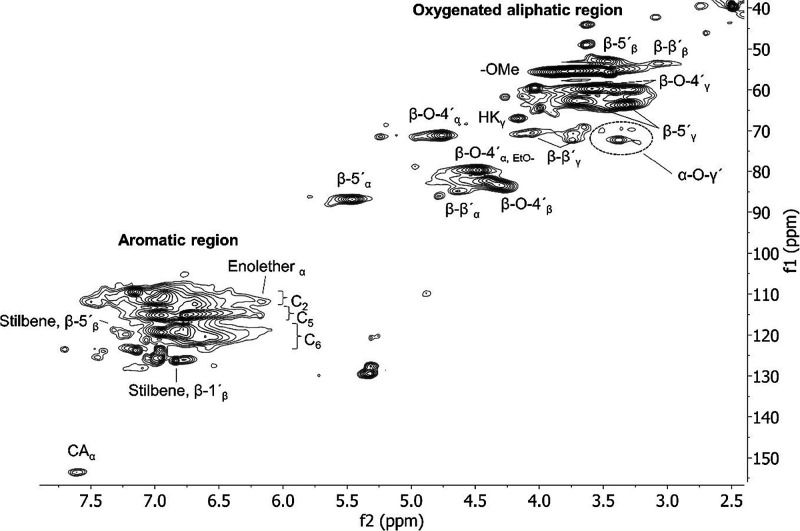
HSQC NMR spectrum of the ethanol-soluble part of the cyclic extracted lignin sample; f1 corresponds to the ^13^C dimension and f2 to the ^1^H dimension. The approximate amount of the main inter-units was β-O-4′ 33, β-5′ 12, and β-β′ 2 per 100 Ar.

Having studied the references, we then studied the biorefinery lignin from the cyclic extraction using the same techniques to assess lignin reactivity and the related mechanisms. The HSQC NMR spectrum of the ethanol fractionated lignin sample, Figure 6, shows the dominance of β-O-4′ at roughly 33 per 100 aromatic rings (Ar). In contrast, the β-O-4′ content of spruce lignin is reported at roughly 50–60%^54^ and that of technical lignins (organosolv and kraft) normally is approximately 10%.^2^ Hence, it seems that the native inter-units are better protected by the cyclic extraction when compared to technical lignin. Other native inter-units detected include β-5′ and β-β′ inter-unit linkages. Slight structural changes occur and include formation of stilbene structures through elimination of formaldehyde from β-1′ and β-5′, and Hibberts ketones from reactions of β-O-4′ structures, all consistent with the literature.^2,46^

The hydroxyl functionality of the lignin sample was quantified by ^31^P NMR, and the spectrum is reported in Figure S11. There was a dominance of aliphatic hydroxyls (3.1 mmol/g lignin) over phenolic hydroxyls (2.2 mmol/g lignin).

**Figure 7:**
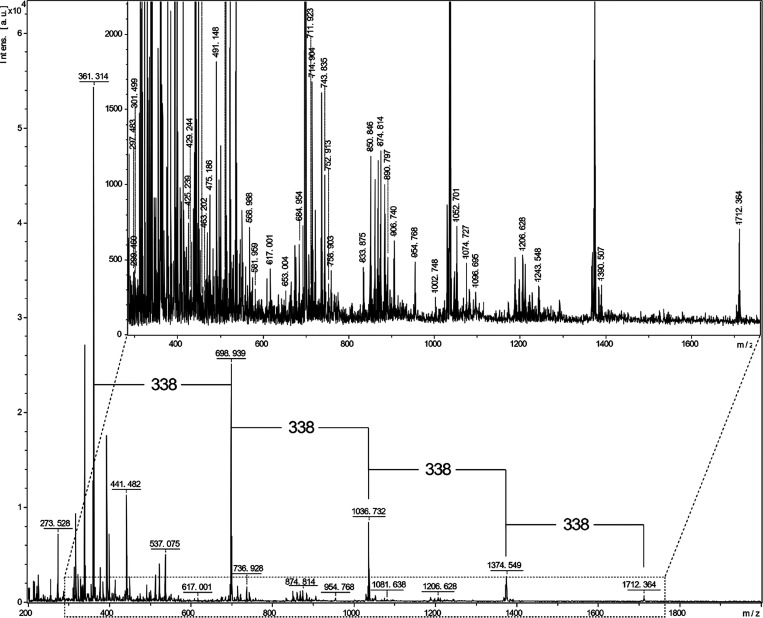
MALDI-TOF MS spectrum of EtOH soluble part of lignin extracted at 160 °C, 1.5 wt % acid, and 70:30 (v/v) ethanol/water.

A MALDI-TOF MS spectrum of the ethanol-soluble lignin sample, analyzed in positive ion mode, is shown in Figure 7. Interestingly, uniformly distanced clusters are observed indicating regular patterns of fragmentation. This fragmentation is a consequence of the organosolv extraction process. Notably, the distances between the clusters indicate a mass increment of 338 Da, in contrast to those observed for the lignin references of between 178 and 196 Da (Figure 3). The 338 Da mass increment might therefore correspond to a dimeric repeating unit resulting from modifications during organosolv process. Typically, masses for unmodified lignin dimeric segments based on the common native inter-units are in the range 360–392 Da. The observed mass difference of 338 Da is therefore consistent with dimeric segments that have lost smaller molecules. Such losses could occur both during lignin extraction and MALDI MS analysis.^56^ Intramolecular condensation reactions are known to occur during the acid catalyzed organosolv extraction process with the formation of stable carbon–carbon bonds.^56^

**Figure 8:**
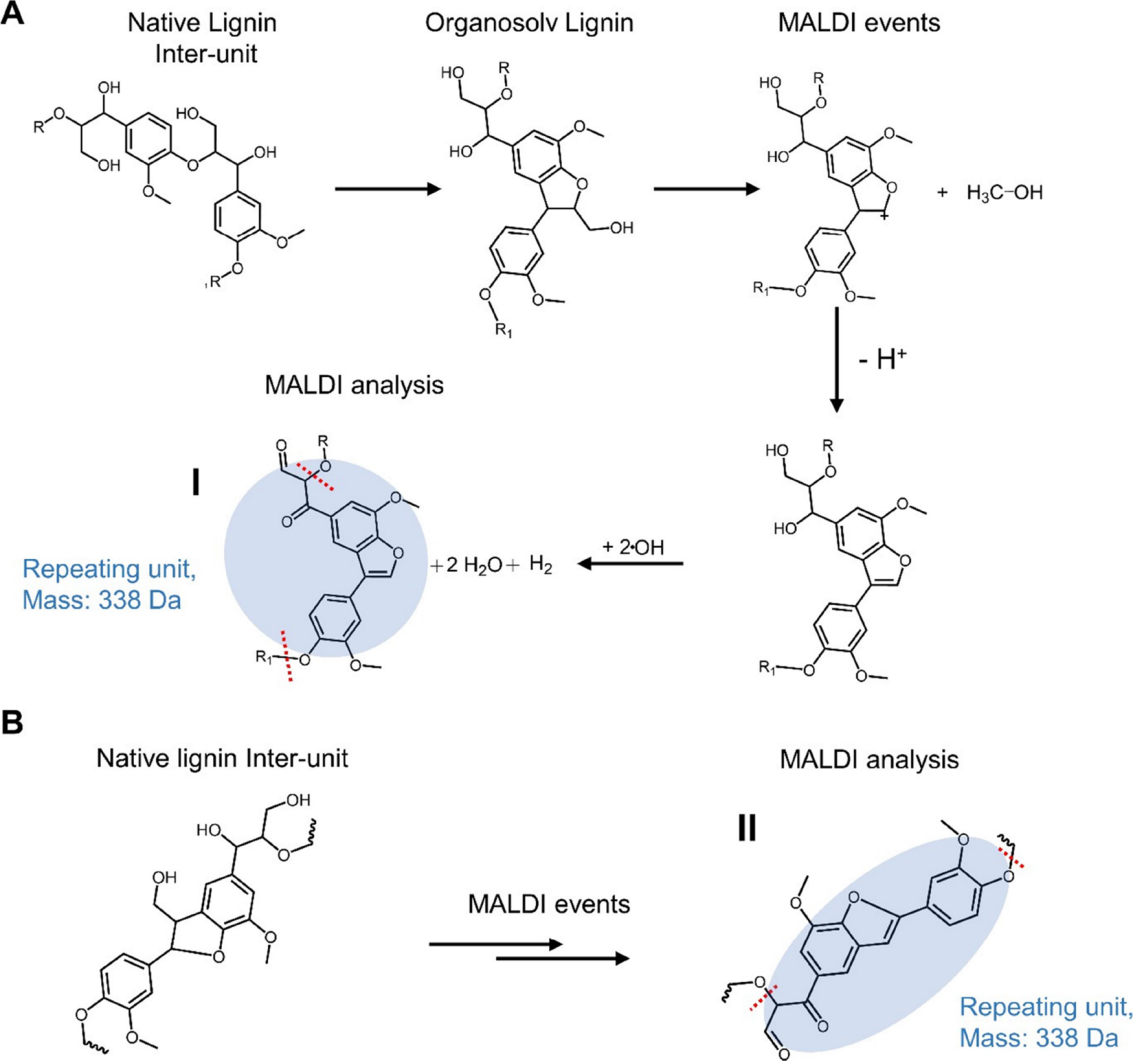
Pathways for the formation of tentative structures from MALDI-TOF MS analysis. The pathyway for the aryl ether is shown in “A”. The pathway for the phenylcoumaran is shown in “B”. The red dotted lines indicate the beginning and end of the repeating unit. There are four dimeric repeating units in each of the structures.

The resultant dimeric repeating units are likely to be connected to each other by ether bonds, which are more easily cleaved during lignin extraction. This provides a reasonable explanation for the uniformly distanced clusters in the MALDI-TOF MS analysis. Accordingly, we propose two structures as repeating units both with a mass of 338 Da. The formation mechanisms are shown in Figure 8. The first structure originates from a β-O-4′ oligomer (repeating units shown in Figure 8A), and the second from an alternating β-O-4′ β-5′ phenyl coumaran oligomer (repeating units shown in Figure 8B). Both of these consist of eight aromatic rings based on the MALDI-TOF MS cluster analysis. These tentative structures are also consistent with the HSQC NMR analysis, which showed that β-O-4′ and β-5′ are the most abundant inter-units in this sample (Figure 6). The proposed modifications of the original structures are consistent with reaction mechanisms during the acid catalyzed organosolv extraction and reasonable events during the MALDI analysis. More specifically, **I** in Figure 8A originates from reactions between two adjacent β-O-4′ sub-units. An intramolecular condensation occurs forming a new linkage through reactive site capping of the benzylic carbocation, formed under the acidic organosolv conditions. The capping reaction forms an α-5′ linkage. Upon MALDI analysis, elimination reactions are proposed due to laser ablation, where methanol is lost through heterolytic cleavage to form a coumaran structure. This loss of methanol in relation to MALDI analysis of lignin has been reported earlier.^56^ The oxidation of the aliphatic hydroxyls to carbonyls as shown could also occur during MALDI analysis. It is reported in the literature that 2,5 DHB is oxidized in MALDI analysis to yield hydroxyl radicals which can initiate further oxidation reactions with substrates.^57^ Here, we propose the formation of water and molecular hydrogen through reactions of hydroxyl radicals with aliphatic hydroxyls to yield ketone- and aldehyde groups in the lignin structure. The intramolecular condensation seen here is beneficial as an internal capping phenomenon when contrasted with intermolecular condensations, which lead to molar mass increase and extraction recalcitrance. Analogues of the intramolecular condensation products have been reported in MALDI MS^56^ and Tandem-MS related studies.^56^ The second structure originating from an alternating β-O-4′ β-5′ sub-structure (Figure 8B) undergoes similar MALDI events to the first to yield a stilbene coumaran structure, **II**.

**Figure 9:**
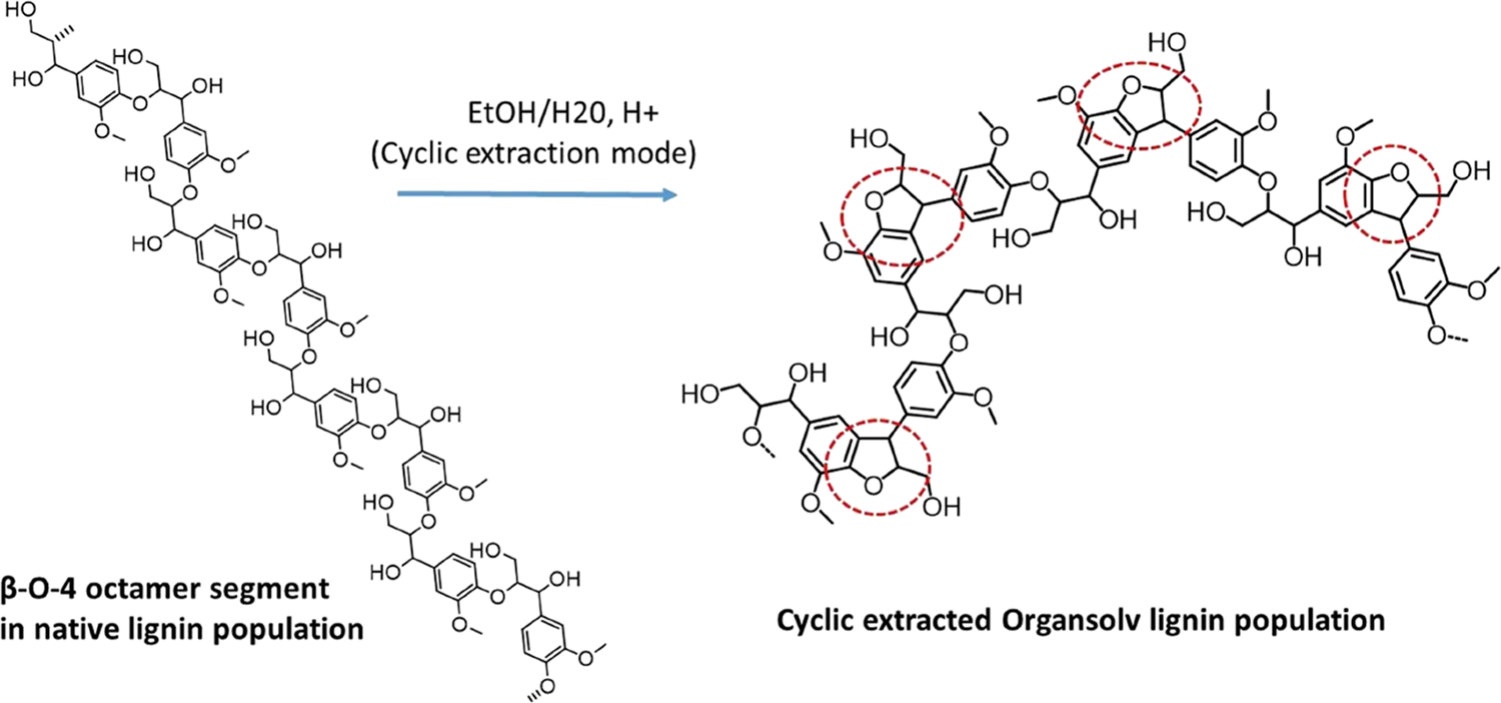
Reactivity of a homogeneous octameric segment consisting of only β-O-4′ linkages in native lignin. An intramolecular condensation is identified that occurs alternately in the β- O-4′ sub-units. The alternate occurrence is marked with red dotted circles.

The MALDI analysis showed that the structural integrity of the lignin oligomers is sufficiently preserved to extract new insights into lignin structure and reactivity. These insights are captured in Figure 9, which is a simplification of Figure 8, and are summarized as follows:

a. The identification of native lignin populations consisting of structurally homogeneous segments. More specifically a population consisting of a segment containing eight aromatic rings coupled by β-O-4′ linkages.
b. Evidence for the occurrence of intramolecular condensation reactions. Although these reactions have previously been reported, the new insight is its occurrence in β-O-4′ sub-units, which intriguingly occurred alternately in the β-O-4′ octameric segment. The reasons for the alternate occurrence were further investigated by DFT studies, now discussed.

### DFT Modeling of Lignin Oligomers Reveals Insights on the Proposed Reactions of Lignin during the Cyclic Organosolv Extraction

**Figure 10:**
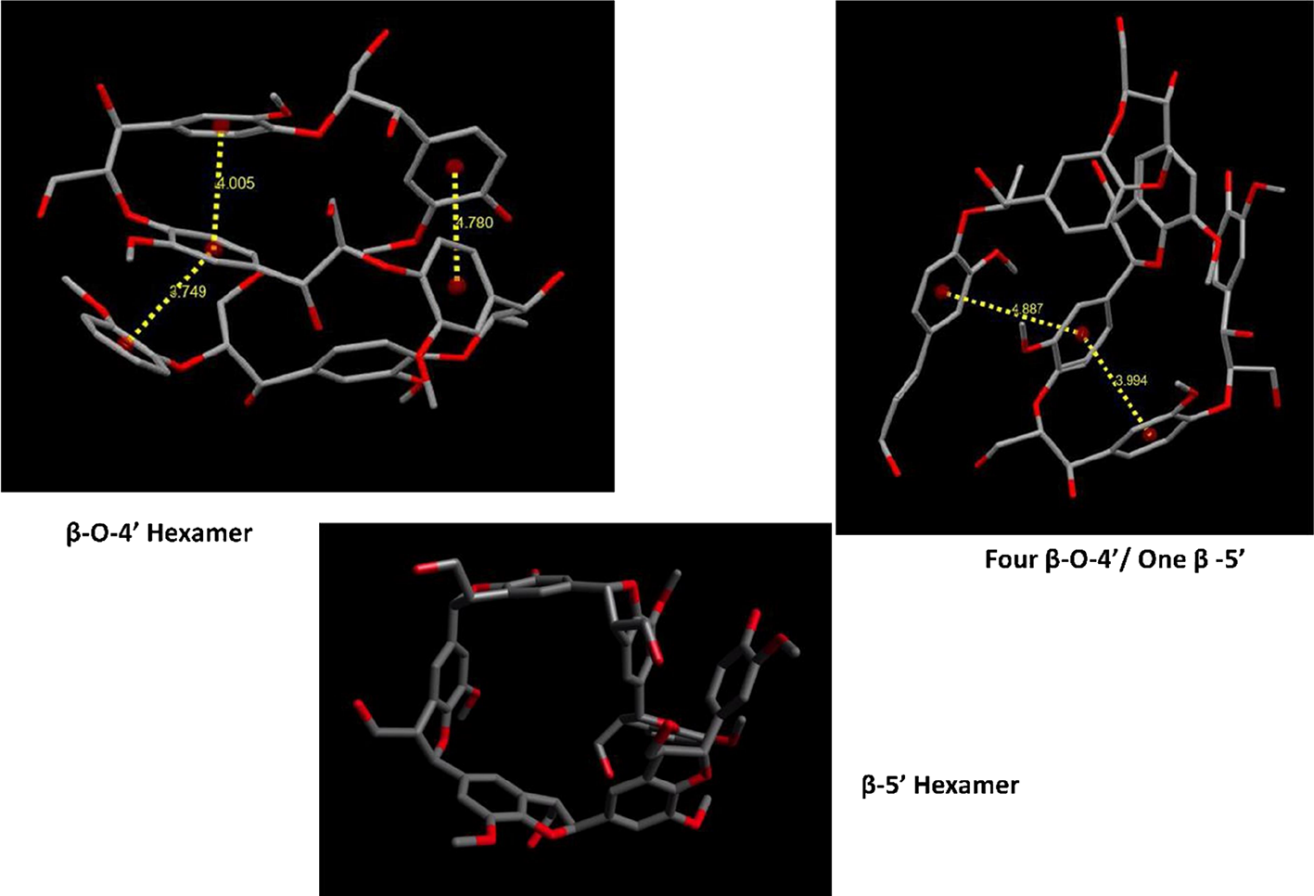
DFT models of the three hexamers. Yellow dotted lines indicate distances between the π–π stacked aromatic rings for both sandwich and T-shape type stacking.

Molecular conformations certainly affect reactions as they determine the accessibility of reactive sites in specific media. In addition, they could provide information on proximity of reactive centers within a molecule. Lignin consists of both stiff and flexible bonds. The β-O-4′ linkage for instance is flexible and can rotate and fold yielding intramolecular π–π stacking. Such stacking has been reported for the middle lamella lignin.^58^ We hypothesized therefore that molecular conformations played important role in the observed alternate condensation reaction shown in Figure 9. To investigate this, DFT experiments were done on three hexamers containing the two most common inter-units from the experimental analyses. The modeling was done on the minimized energy conformers in DMSO and ethanol as the solvents. Interestingly, no changes in conformations are observed except for small changes in the molecular volumes. The models produced using ethanol as the solvent are presented in Figure 10. Top left consists only of β-O-4’s, top right of four β-O-4′ s with a β-5′ phenylcoumaran inter-unit in the middle, and the bottom only β-5′ phenylcoumarans.

As observed, π–π stacking occurs and the role of β-O-4’s is essential. In the β-O-4′ only hexamer, two regions were observed; the first consisting of sandwiched stacked aromatic rings and the second of T-shape stacking with the distances between the sandwich stacks in the range 3–4 Å and that between the T-shaped stacks in the range 4–5 Å. The mixed composition hexamer β-O-4′/β-5′ on the other hand showed longer distances between the sandwich stacking in the range 4–5 Å, indicating some constraints to closer sandwich stacking. The specific type of stacking, sandwich or T-shaped, plays a role in the molecular conformation and depends on the inter-unit linkages and bonding sequences. The absence of β-O-4’s, for instance, as seen in the β-5′-phenylcoumaran hexamer, yields a ring formation. Notably, the hydroxyls are exposed outwardly for all the models. This may explain why the aliphatic alcohols are easy targets for the oxidation reactions observed in the MALDI MS analysis of the biorefinery lignin.

**Figure 11:**
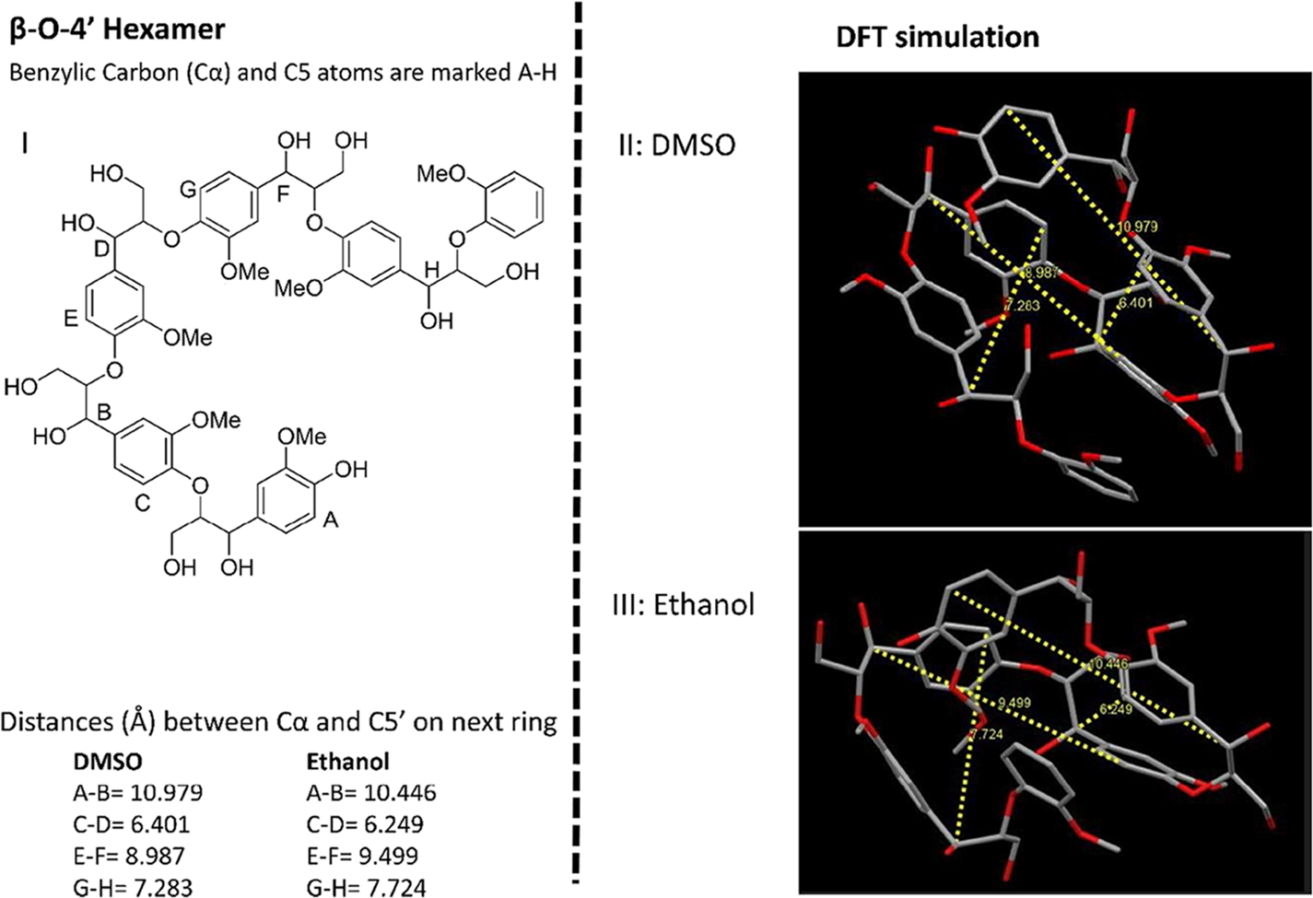
Left: The hexamer motif used for the DFT simulation, I. Right: Distances between the benzylic carbon and 5′ on the next aromatic ring, marked with the yellow dotted lines on the simulations. DMSO and ethanol as solvent systems studied.

The β-O-4′ containing oligomers form a spiral molecule, due to the π–π stacking, and this plays a key role in bringing reactive centers of relevance to the intramolecular condensation reactions (Figure 9) and may explain why the intramolecular condensation reactions occurred alternately in the β-O-4′ oligomer, Figure 9. Since proximity is a criterion for reactions between two sites, the distances between the benzylic carbon (C-alpha) and C5′ on the next aromatic ring were measured from the calculations as shown in Figure 11. Clearly, this distance is shorter for sandwich stacked rings when compared to T-shaped stacked rings. The proximity of reactive sites is therefore determined by the presence and type of stacking. As observed, the stacking in the β-O-4′ oligomer occurs alternately between sandwich and T-shaped and provides a plausible explanation for the alternate occurrence of condensation between aromatic systems shown in the proposed structure (Figure 9, **I**). This supports the hypothesis and provides a new insight into the role of intramolecular π–π stacking on lignin reactivity. Interestingly, the precise molecular structure determines the type of stacking, which in turn dictates the proximity of reactive sites.

## Conclusions

Lignin valorization efforts are currently limited by the availability of high-quality lignins. New lignin biorefinery concepts are therefore currently being investigated. This work concerns fundamental insights into the structure of lignin obtained from a new lignin biorefining process that adopts a physical protection principle. The approach used in the study was to contrast the structures of lignin synthesized by mimicking native lignin polymerization chemistry (reference for native lignin) with that of the biorefinery lignin. For this purpose, a combination of NMR, MALDI-TOF MS, and DFT were used as analytical tools. MALDI-TOF MS was successfully shown to complement state-of-the-art lignin analysis by HSQC NMR. More specifically, lignin molecular populations were studied to reveal insights into bio-mimicked lignin polymerization as well as reactivity of lignin during organosolv extraction performed in a cyclic mode. HSQC NMR analysis of the bulk bio-mimicked lignin showed the abundance of aryl ether linkages (β-O-4′) while the linkage sequence in the molecular populations was unraveled by MALDI MS analysis. The emergence of etherified branching points in lignin was identified at the tetramer stage of the polymerization process.

Analysis of the organosolv biorefinery lignin by HSQC NMR showed an abundance of aryl ether linkages (β-O-4′), confirming that physical protection indeed occurred due to performing the extraction in cyclic mode. MALDI MS analysis of the same revealed a population of uniform oligomers consisting of eight aromatic rings with dimeric repeating units. The tentative structures proposed from the MALDI MS analysis indicate the occurrence of intramolecular condensation reactions during the extraction process. The tentative lignin structures are proposed to originate from β-O-4′ octamers and/or alternating β-O-4′ β-5 tetramers. DFT simulations revealed the role that intramolecular π–π stacking of aromatic rings (sandwich or T- shaped) plays in the condensation reaction. More specifically, the proximity of reactive sites was generally improved through π–π stacking but more so for sandwich π–π stacking when contrasted with T-shape π–π stacking.

The combination of complementary chemical analyses, together with theoretical modeling as shown here, is particularly useful for fundamental studies on lignin. Although the MALDI MS analysis may not be fully representative of the lignin sample due to discrimination in crystallization and ionization, this study demonstrates the usefulness of combining NMR, MALDI MS, and DFT simulations for the study of lignin.

## Supporting Information Available

Molar mass determination of lignin; analysis of aryl ethers; analysis of phenyl coumaran; analysis of pinresinol; analysis of the lignin reference and matrix; analysis of extracted lignin, spot 1; analysis of extracted lignin, spot 2; analysis of coniferyl alcohol; characterization of aryl ethers; characterization of phenyl coumaran; characterization of pinresinol; analysis and quantification of hydroxyl functionalities of the extracted lignin; and illustration of lignin polymerization (PDF)

## References

[ref1] Ragauskas A. J.; Beckham G. T.; Biddy M. J.; Chandra R.; Chen F.; Davis M. F.; Davison B. H.; Dixon R. A.; Gilna P.; Keller M.; Langan P.; Naskar A. K.; Saddler J. N.; Tschaplinski T. J.; Tuskan G. A.; Wyman C. E. Lignin valorization: improving lignin processing in the biorefinery. Science 2014, 344, 1246843 10.1126/science.1246843. 24833396

[ref2] Karlsson M.; Vegunta V. L.; Deshpande R.; Lawoko M. Protected lignin biorefining through cyclic extraction: gaining fundamental insights into the tuneable properties of lignin by chemometrics (vol 24, pg 1211, 2022). Green Chem. 2022, 24, 1211–1223. 10.1039/D1GC04171A.

[ref3] Luo X.; Li Y.; Gupta N. K.; Sels B.; Ralph J.; Shuai L. Protection strategies enable selective conversion of biomass. Angew. Chem., Int. Ed. 2020, 59, 11704–11716. 10.1002/anie.201914703. 32017337

[ref4] Deuss P. J.; Barta K.; de Vries J. G. Homogeneous catalysis for the conversion of biomass and biomass-derived platform chemicals. Catal. Sci. Technol. 2014, 4, 1174–1196. 10.1039/C3CY01058A.

[ref5] Li Y.; Demir B.; Vázquez Ramos L. M.; Chen M.; Dumesic J. A.; Ralph J. Kinetic and mechanistic insights into hydrogenolysis of lignin to monomers in a continuous flow reactor. Green Chem. 2019, 21, 3561–3572. 10.1039/C9GC00986H.

[ref6] Shuai L.; Amiri M. T.; Questell-Santiago Y. M.; Héroguel F.; Li Y.; Kim H.; Meilan R.; Chapple C.; Ralph J.; Luterbacher J. S. Formaldehyde stabilization facilitates lignin monomer production during biomass depolymerization. Science 2016, 354, 329–333. 10.1126/science.aaf7810. 27846566

[ref7] Lan W.; Amiri M. T.; Hunston C. M.; Luterbacher J. S. Protection group effects during α, γ-diol lignin stabilization promote high-selectivity monomer production. Angew. Chem., Int. Ed. 2018, 130, 1370–1374. 10.1002/ange.201710838. 29210487

[ref8] Vendamme R.; Behaghel de Bueren J.; Gracia-Vitoria J.; Isnard F.; Mulunda M. M.; Ortiz P.; Wadekar M.; Vanbroekhoven K.; Wegmann C.; Buser R.; Héroguel F.; Luterbacher J. S.; Eevers W. Aldehyde-Assisted Lignocellulose Fractionation Provides Unique Lignin Oligomers for the Design of Tunable Polyurethane Bioresins. Biomacromolecules 2020, 21, 4135–4148. 10.1021/acs.biomac.0c00927. 32845140

[ref9] Zijlstra D. S.; de Korte J.; de Vries E. P.; Hameleers L.; Wilbers E.; Jurak E.; Deuss P. J. Highly efficient semi-continuous extraction and in-line purification of high β-O- 4 butanosolv lignin. Front. Chem. 2021, 9, 655983 10.3389/fchem.2021.655983. 34041222 PMC8141753

[ref10] Balakshin M.; Capanema E. A.; Zhu X.; Sulaeva I.; Potthast A.; Rosenau T.; Rojas O. J. Spruce milled wood lignin: linear, branched or cross-linked?. Green Chem. 2020, 22, 3985–4001. 10.1039/D0GC00926A.

[ref11] Crestini C.; Melone F.; Sette M.; Saladino R. Milled wood lignin: a linear oligomer. Biomacromolecules 2011, 12, 3928–3935. 10.1021/bm200948r. 21928799

[ref12] Sapouna I.; Lawoko M. Deciphering lignin heterogeneity in ball milled softwood: unravelling the synergy between the supramolecular cell wall structure and molecular events. Green Chem. 2021, 23, 3348–3364. 10.1039/D0GC04319B.

[ref13] Lancefield C. S.; Wienk H. L. J.; Boelens R.; Weckhuysen B. M.; Bruijnincx P. C. A. Identification of a diagnostic structural motif reveals a new reaction intermediate and condensation pathway in kraft lignin formation. Chem. Sci. 2018, 9, 6348–6360. 10.1039/C8SC02000K. 30310563 PMC6115679

[ref14] Crestini C.; Lange H.; Sette M.; Argyropoulos D. S. On the structure of softwood kraft lignin. Green Chem. 2017, 19, 4104–4121. 10.1039/C7GC01812F.

[ref15] Giummarella N.; Lindén P. R. A.; Areskogh D.; Lawoko M. Fractional profiling of kraft lignin structure: Unravelling insights on lignin reaction mechanisms. ACS Sustainable Chem. Eng. 2020, 8, 1112–1120. 10.1021/acssuschemeng.9b06027.

[ref16] Morreel K.; Dima O.; Kim H.; Lu F.; Niculaes C.; Vanholme R.; Dauwe R.; Goeminne G.; Inzé D.; Messens E.; Ralph J.; Boerjan W. Mass Spectrometry-Based Sequencing of Lignin Oligomers. Plant Physiol. 2010, 153, 1464–1478. 10.1104/pp.110.156489. 20554692 PMC2923877

[ref17] Sheng H.; Tang W.; Gao J.; Riedeman J. S.; Li G.; Jarrell T. M.; Hurt M. R.; Yang L.; Murria P.; Ma X.; Nash J. J.; Kenttämaa H. I. (−)ESI/CAD MSn Procedure for Sequencing Lignin Oligomers Based on a Study of Synthetic Model Compounds with β-O-4 and 5-5 Linkages. Anal. Chem. 2017, 89, 13089–13096. 10.1021/acs.analchem.7b01911. 29116757

[ref18] Prothmann J.; Spégel P.; Sandahl M.; Turner C. Identification of lignin oligomers in Kraft lignin using ultra-high-performance liquid chromatography/high-resolution multiple- stage tandem mass spectrometry (UHPLC/HRMSn). Anal. Bioanal. Chem. 2018, 410, 7803–7814. 10.1007/s00216-018-1400-4. 30306235 PMC6244760

[ref19] Prothmann J.; Sun M.; Spégel P.; Sandahl M.; Turner C. Ultra-high-performance supercritical fluid chromatography with quadrupole-time-of-flight mass spectrometry (UHPSFC/QTOF-MS) for analysis of lignin-derived monomeric compounds in processed lignin samples. Anal. Bioanal. Chem. 2017, 409, 7049–7061. 10.1007/s00216-017-0663-5. 29030670 PMC5717129

[ref20] Sun M.; Sandahl M.; Turner C. Comprehensive on-line two-dimensional liquid chromatography × supercritical fluid chromatography with trapping column-assisted modulation for depolymerised lignin analysis. J. Chromatogr. A 2018, 1541, 21–30. 10.1016/j.chroma.2018.02.008. 29452928

[ref21] Zhang J.; Jiang Y.; Easterling L. F.; Anstner A.; Li W.; Alzarieni K. Z.; Dong X.; Bozell J.; Kenttämaa H. I. Compositional analysis of organosolv poplar lignin by using high-performance liquid chromatography/high-resolution multi-stage tandem mass spectrometry. Green Chem. 2021, 23, 983–1000. 10.1039/D0GC03398G.

[ref22] Prothmann J.; Li K.; Hulteberg C.; Spégel P.; Sandahl M.; Turner C. Nontargeted Analysis Strategy for the Identification of Phenolic Compounds in Complex Technical Lignin Samples. ChemSusChem 2020, 13, 4605–4612. 10.1002/cssc.202000951. 32468723 PMC7540015

[ref23] Metzger J. O.; Bicke C.; Faix O.; Tuszynski W.; Angermann R.; Karas M.; Strupat K. Matrix-assisted laser desorption mass spectrometry of lignins. Angew. Chem., Int. Ed. Engl. 1992, 31, 762–764. 10.1002/anie.199207621.

[ref24] Cameron A.; Eggers D. Jr. An Ion “Velocitron”. Rev. Sci. Instrum. 1948, 19, 605–607. 10.1063/1.1741336.

[ref25] Mamyrin B.; Karataev V.; Shmikk D.; Zagulin V. The mass-reflectron, a new nonmagnetic time-of-flight mass spectrometer with high resolution. Zh. Eksp. Teor. Fiz. 1973, 64, 82–89.

[ref26] Trimpin S.; Räder H. J.; Müllen K. Investigations of theoretical principles for MALDI-MS derived from solvent-free sample preparation: Part I. Preorganization. Int. J. Mass Spectrom. 2006, 253, 13–21. 10.1016/j.ijms.2005.10.008.

[ref27] Kosyakov D. S.; Anikeenko E. A.; Ul’yanovskii N. V.; Khoroshev O. Y.; Shavrina S.; Gorbova N. S. Ionic liquid matrices for MALDI mass spectrometry of lignin. Anal. Bioanal. Chem. 2018, 410, 7429–7439. 10.1007/s00216-018-1353-7. 30229310

[ref28] Qi Y.; Volmer D. A. Chemical diversity of lignin degradation products revealed by matrix-optimized MALDI mass spectrometry. Anal. Bioanal. Chem. 2019, 411, 6031–6037. 10.1007/s00216-019-01984-y. 31278551

[ref29] Letourneau D. R.; Volmer D. A. Mass spectrometry-based methods for the advanced characterization and structural analysis of lignin: A review. Mass Spectrom. Rev. 2023, 42, 144–188. 10.1002/mas.21716. 34293221

[ref30] Richel A.; Vanderghem C.; Simon M.; Wathelet B.; Paquot M. Evaluation of matrix-assisted laser desorption/ionization mass spectrometry for second-generation lignin analysis. Anal. Chem. Insights 2012, 7, 79–89. 10.4137/ACI.S10799. 23300342 PMC3528113

[ref31] Yoshioka K.; Ando D.; Watanabe T. A Comparative Study of Matrix- and Nano- assisted Laser Desorption/Ionisation Time-of-Flight Mass Spectrometry of Isolated and Synthetic Lignin. Phytochem. Anal. 2012, 23, 248–253. 10.1002/pca.1350. 21898628

[ref32] Shigeto J.; Honjo H.; Fujita K.; Tsutsumi Y. Generation of lignin polymer models via dehydrogenative polymerization of coniferyl alcohol and syringyl alcohol via several plant peroxidases involved in lignification and analysis of the resulting DHPs by MALDI-TOF analysis. Holzforschung 2018, 72, 267–274. 10.1515/hf-2017-0125.

[ref33] Kim S.; Chmely S. C.; Nimlos M. R.; Bomble Y. J.; Foust T. D.; Paton R. S.; Beckham G. T. Computational study of bond dissociation enthalpies for a large range of native and modified lignins. J. Phys. Chem. Lett. 2011, 2, 2846–2852. 10.1021/jz201182w.

[ref34] Parthasarathi R.; Romero R. A.; Redondo A.; Gnanakaran S. Theoretical study of the remarkably diverse linkages in lignin. J. Phys. Chem. Lett. 2011, 2, 2660–2666. 10.1021/jz201201q.

[ref35] Younker J. M.; Beste A.; Buchanan A. III Computational study of bond dissociation enthalpies for lignin model compounds: β-5 arylcoumaran. Chem. Phys. Lett. 2012, 545, 100–106. 10.1016/j.cplett.2012.07.017.

[ref36] Elder T.; Beste A. Density functional theory study of the concerted pyrolysis mechanism for lignin models. Energy Fuels 2014, 28, 5229–5235. 10.1021/ef5013648.

[ref37] Houston R. W.; Elder T. J.; Abdoulmoumine N. H. Investigation into the Pyrolysis Bond Dissociation Enthalpies (BDEs) of a Model Lignin Oligomer Using Density Functional Theory (DFT). Energy Fuels 2022, 36, 1565–1573. 10.1021/acs.energyfuels.1c03238.

[ref38] Azad T.; Torres H. F.; Auad M. L.; Elder T.; Adamczyk A. J. Isolating key reaction energetics and thermodynamic properties during hardwood model lignin pyrolysis. Phys. Chem. Chem. Phys. 2021, 23, 20919–20935. 10.1039/D1CP02917G. 34541592

[ref39] Azad T.; Schuler J. D.; Auad M. L.; Elder T.; Adamczyk A. J. Model Lignin Oligomer Pyrolysis: Coupled Conformational and Thermodynamic Analysis of β-O-4′ Bond Cleavage. Energy Fuels 2020, 34, 9709–9724. 10.1021/acs.energyfuels.0c01573.

[ref40] Bock P.; Nousiainen P.; Elder T.; Blaukopf M.; Amer H.; Zirbs R.; Potthast A.; Gierlinger N. Infrared and Raman spectra of lignin substructures: Dibenzodioxocin. J. Raman Spectrosc. 2020, 51, 422–431. 10.1002/jrs.5808. 32214622 PMC7079546

[ref41] Berstis L.; Elder T.; Crowley M.; Beckham G. Radical Nature of C-Lignin. ACS Sustainable Chem. Eng. 2016, 4, 5327–5335. 10.1021/acssuschemeng.6b00520.

[ref42] Elder T.; del Rio J. C.; Ralph J.; Rencoret J.; Kim H.; Beckham G. T.; Crowley M. F. Coupling and reactions of lignols and new lignin monomers: A density functional theory study. ACS Sustainable Chem. Eng. 2020, 8, 11033–11045. 10.1021/acssuschemeng.0c02880.

[ref43] Elder T.; Carlos del Río J.; Ralph J.; Rencoret J.; Kim H.; Beckham G. T. Radical coupling reactions of piceatannol and monolignols: A density functional theory study. Phytochemistry 2019, 164, 12–23. 10.1016/j.phytochem.2019.04.003. 31060026

[ref44] Elder T.; Rencoret J.; Del Río J. C.; Kim H.; Ralph J. Radical coupling reactions of hydroxystilbene glucosides and coniferyl alcohol: a density functional theory study. Front. Plant Sci. 2021, 12, 642848 10.3389/fpls.2021.642848. 33737945 PMC7960926

[ref45] Zhao Y.; Truhlar D. G. The M06 suite of density functionals for main group thermochemistry, thermochemical kinetics, noncovalent interactions, excited states, and transition elements: two new functionals and systematic testing of four M06-class functionals and 12 other functionals. Theor. Chem. Acc. 2008, 120, 215–241. 10.1007/s00214-007-0310-x.

[ref46] Karlsson M.; Giummarella N.; Lindén P. A.; Lawoko M. Toward a Consolidated Lignin Biorefinery: Preserving the Lignin Structure through Additive-Free Protection Strategies. ChemSusChem 2020, 13, 4666–4677. 10.1002/cssc.202000974. 32530110 PMC7540675

[ref47] Gellerstedt G. Gel permeation chromatography. In Methods in lignin chemistry, Springer: 1992; pp. 487–497.

[ref48] Argyropoulos D. 31 P NMR in wood chemistry: A review of recent progress. Res. Chem. Intermed. 1995, 21, 373 10.1007/BF03052265.

[ref49] Granata A.; Argyropoulos D. S. 2-Chloro-4, 4, 5, 5-tetramethyl-1, 3, 2- dioxaphospholane, a reagent for the accurate determination of the uncondensed and condensed phenolic moieties in lignins. J. Agric. Food Chem. 1995, 43, 1538–1544. 10.1021/jf00054a023.

[ref50] Kim H.; Ralph J. Simplified Preparation of Coniferyl and Sinapyl Alcohols. J. Agric. Food Chem. 2005, 53, 3693–3695. 10.1021/jf047787n. 15853421

[ref51] Lu F.; Ralph J. Highly Selective Syntheses of Coniferyl and Sinapyl Alcohols. J. Agric. Food Chem. 1998, 46, 1794–1796. 10.1021/jf970953p.

[ref52] Jawerth M.; Lawoko M.; Lundmark S.; Perez-Berumen C.; Johansson M. Allylation of a lignin model phenol: a highly selective reaction under benign conditions towards a new thermoset resin platform. RSC Adv. 2016, 6, 96281–96288. 10.1039/C6RA21447A.

[ref53] Lancefield C. S.; Westwood N. J. The synthesis and analysis of advanced lignin model polymers. Green Chem. 2015, 17, 4980–4990. 10.1039/C5GC01334H.

[ref54] Ralph J.; Lapierre C.; Boerjan W. Lignin structure and its engineering. Curr. Opin. Biotechnol. 2019, 56, 240–249. 10.1016/j.copbio.2019.02.019. 30921563

[ref55] Ralph J.; Lundquist K.; Brunow G.; Lu F.; Kim H.; Schatz P. F.; Marita J. M.; Hatfield R. D.; Ralph S. A.; Christensen J. H.; Boerjan W. Lignins: natural polymers from oxidative coupling of 4-hydroxyphenyl-propanoids. Phytochem. Rev. 2004, 3, 29–60. 10.1023/B:PHYT.0000047809.65444.a4.

[ref56] Dababi I.; Gimello O.; Elaloui E.; Quignard F.; Brosse N. Organosolv lignin- based wood adhesive. Influence of the lignin extraction conditions on the adhesive performance. Polymers 2016, 8, 340 10.3390/polym8090340. 30974615 PMC6431968

[ref57] McAvey K. M.; Guan B.; Fortier C. A.; Tarr M. A.; Cole R. B. Laser-induced oxidation of cholesterol observed during MALDI-TOF mass spectrometry. J. Am. Soc. Mass Spectrom. 2011, 22, 659–669. 10.1007/s13361-011-0074-3. 21472605

[ref58] Terashima N.; Yoshida M.; Hafrén J.; Fukushima K.; Westermark U. Proposed supramolecular structure of lignin in softwood tracheid compound middle lamella regions. Holzforschung 2012, 66, 907–915. 10.1515/hf-2012-0021.

