# Supplementary material for: Lignin Structure and Reactivity in the Organosolv Process Studied by NMR Spectroscopy, Mass Spectrometry, and Density Functional Theory
Source: Biomacromolecules. 2023 Apr 20;24(5):2314–26. doi: 10.1021/acs.biomac.3c00186 (PMC10170516; doi:10.1021/acs.biomac.3c00186)
Supplement: Supplementary file 1 — bm3c00186_si_001.pdf [file bm3c00186_si_001.pdf]

Supporting information

Lignin Structure and Reactivity in Organosolv process Studied by the NMR Spectroscopy, Mass Spectrometry and Density Functional Theory

Maria Karlsson,[a,d] Joakim Romson,[b] Thomas Elder,[c] Åsa Emmer*,[b] and Martin Lawoko* [a,d]

[a] Wallenberg Wood Science Center, Department of Fiber and Polymer Technology, School of Chemistry, Biotechnology and Health, KTH Royal Institute of Technology, Teknikringen 56-58, SE-100 44 Stockholm, Sweden.

[b] Analytical Chemistry, Division of Applied Physical Chemistry, Department of Chemistry, School of Engineering Sciences in Chemistry, Biotechnology and Health, Royal Institute of Technology, KTH Teknikringen 36, SE-100 44 Stockholm, Sweden.

[c] USDA-Forest Service, Southern Research Station, 521 Devall Drive, Auburn, AL, 36849, USA.

[d] Division of Wood Chemistry and Pulp Technology, Department of Fiber and Polymer Technology, School of Chemistry, Biotechnology and Health, KTH Royal Institute of Technology, Teknikringen 56-58, SE-100 44 Stockholm, Sweden.

Contents

1. SEC 1

Molar mass parameters of unfractionated lignin 1

2. MALDI-TOF MS 2

2.1 Lignin dimeric references 2

2.2 Lignin oligomeric references 4

2.3 Extracted spruce lignin, ethanol soluble fraction 5

3. NMR spectra 6

3.1 ^{1}H NMR 6

3.2 HSQC NMR of the separated dimer references 6

3.3 ^{31}P NMR of extracted lignin 8

References 9

1. SEC

Molar mass parameters of unfractionated lignin

Table S1. The Molar mass parameters of unfractionated lignin and the insoluble ethanol fraction of the lignin.

Lignin sample	Mn	Mw	DP_n	Đ
Unfractionated cyclic lignin	1800	7100	10	3.9
EtOH insoluble cyclic lignin	3900	11900	22	3.0

2. MALDI-TOF MS

2.1 Lignin dimeric references

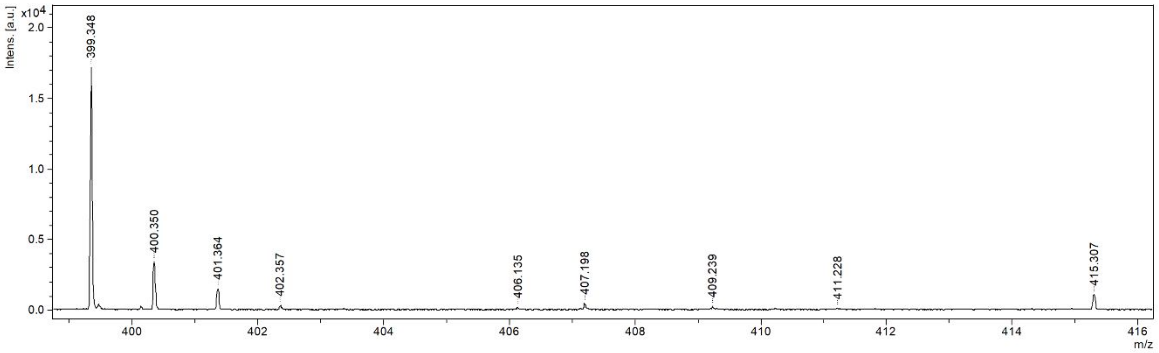

Figure S1. MALDI-TOF MS spectrum of β-O-4′ dimer, with the sodium adduct ion detected at *m/z* 399 and the potassium adduct ion at *m/z* 415.

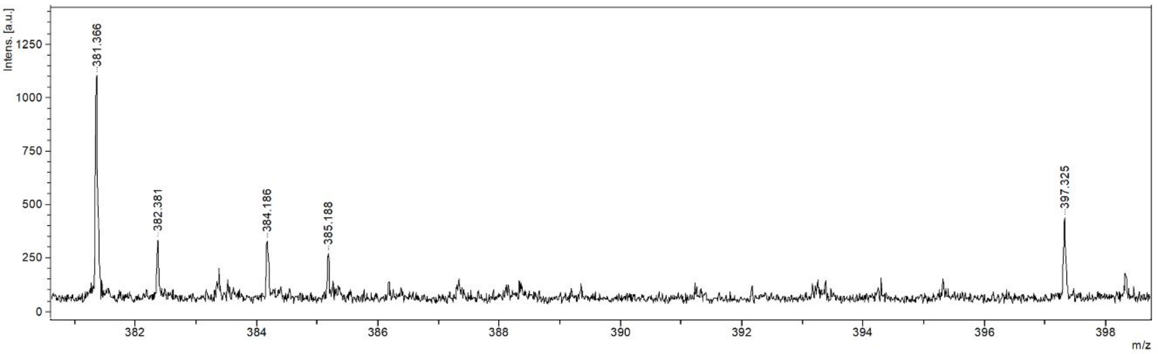

Figure S2. MALDI-TOF MS spectrum of β-5′ dimer, with the sodium adduct ion detected at *m/z* 381 and the potassium adduct ion at *m/z* 397.

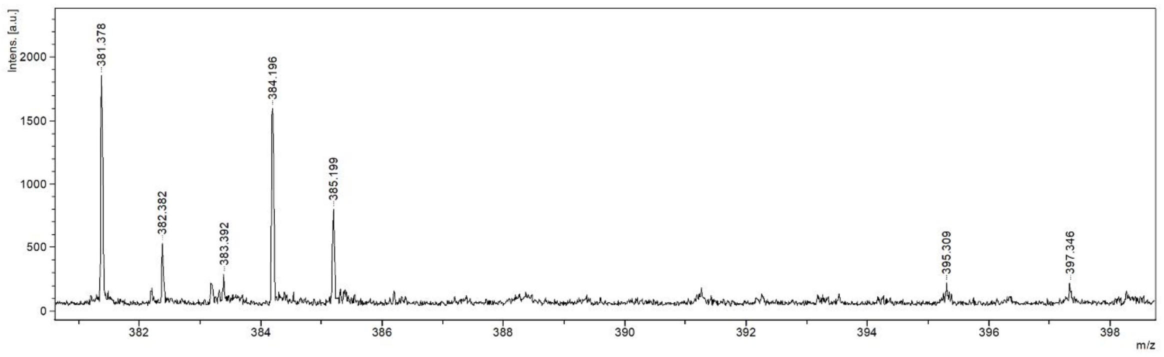

Figure S3. MALDI-TOF MS spectrum of β-β′ dimer, with the sodium adduct ion detected at *m/z* 381 and the potassium adduct ion at *m/z* 397.

2.2 Lignin oligomeric references

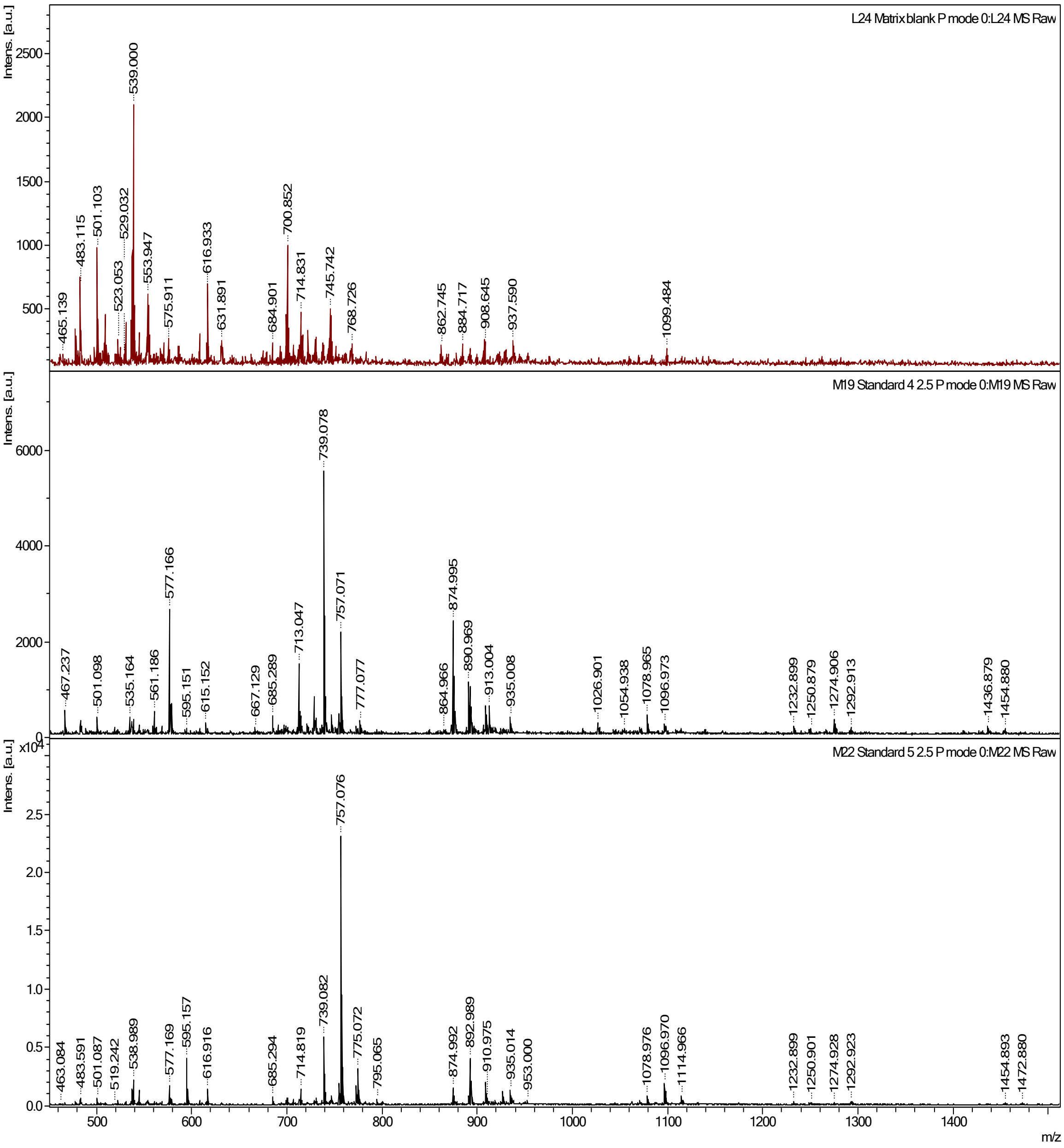

Figure S4. MALDI-TOF MS spectra of the oligomeric reference and matrix (blank).

2.3 Extracted spruce lignin, ethanol soluble fraction

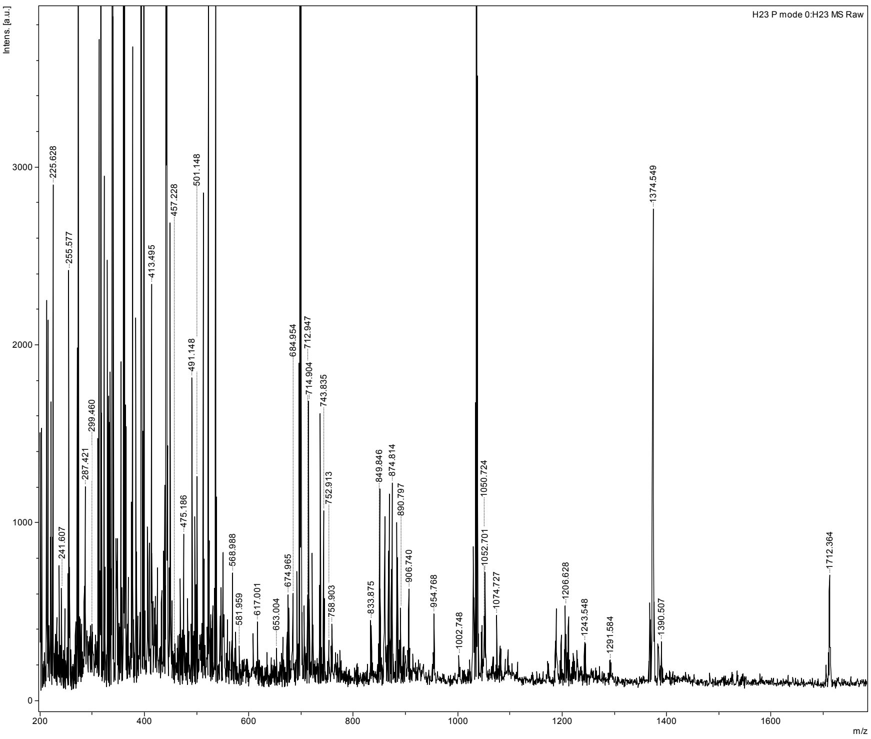

Figure S5. MALDI-TOF MS spectrum of the ethanol soluble fraction of cyclic extracted lignin, spot 1.

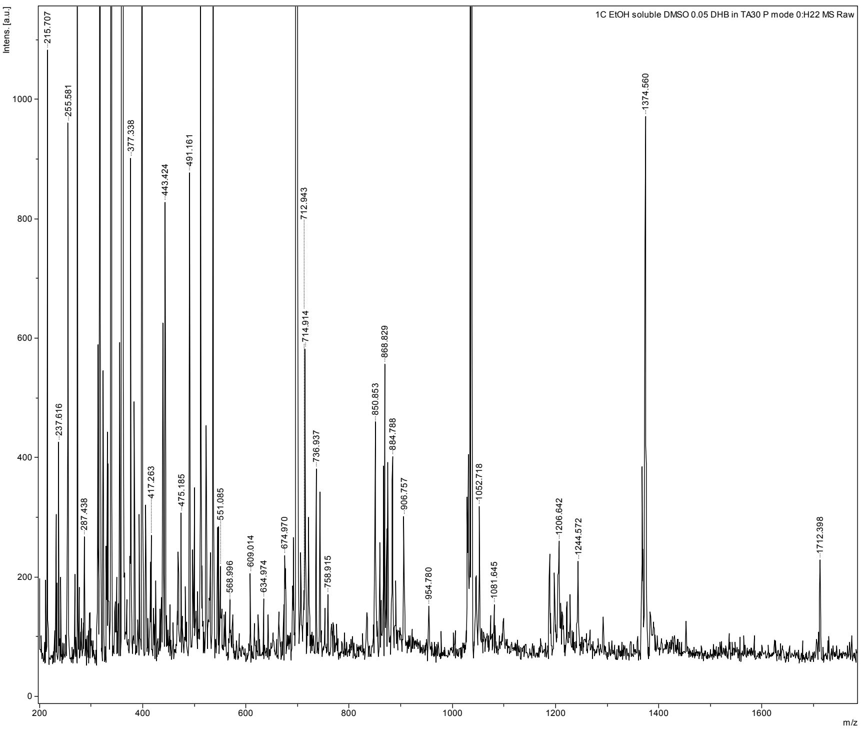

Figure S6. MALDI-TOF MS spectrum of the ethanol soluble fraction of cyclic extracted lignin, spot 2.

3. NMR spectra

3.1 ¹H NMR

Reduction of coniferyl aldehyde to coniferyl alcohol.

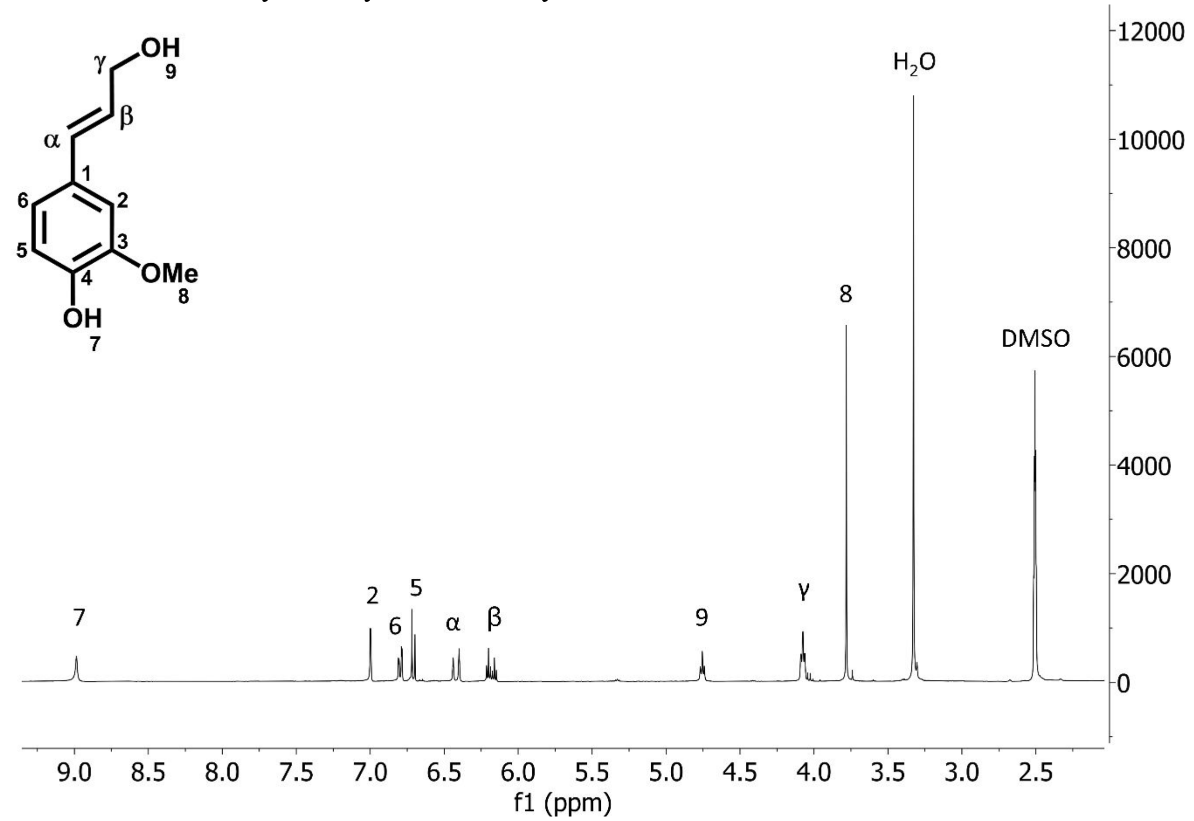

Figure S7. The coniferyl alcohol product obtained after the reduction. The assignment is in accordance with assignment found in literature.[1, 2]

3.2 HSQC NMR of the separated dimer references

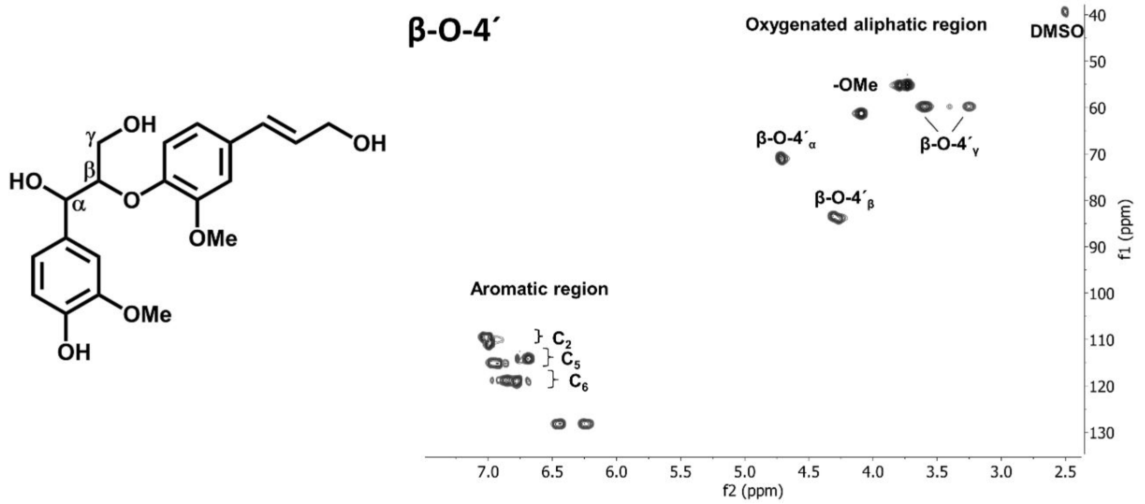

Figure S8. HSQC NMR spectrum of the β-O-4′ dimer, where f1 corresponds to the ^{13}C dimension and f2 to the ^{1}H dimension.

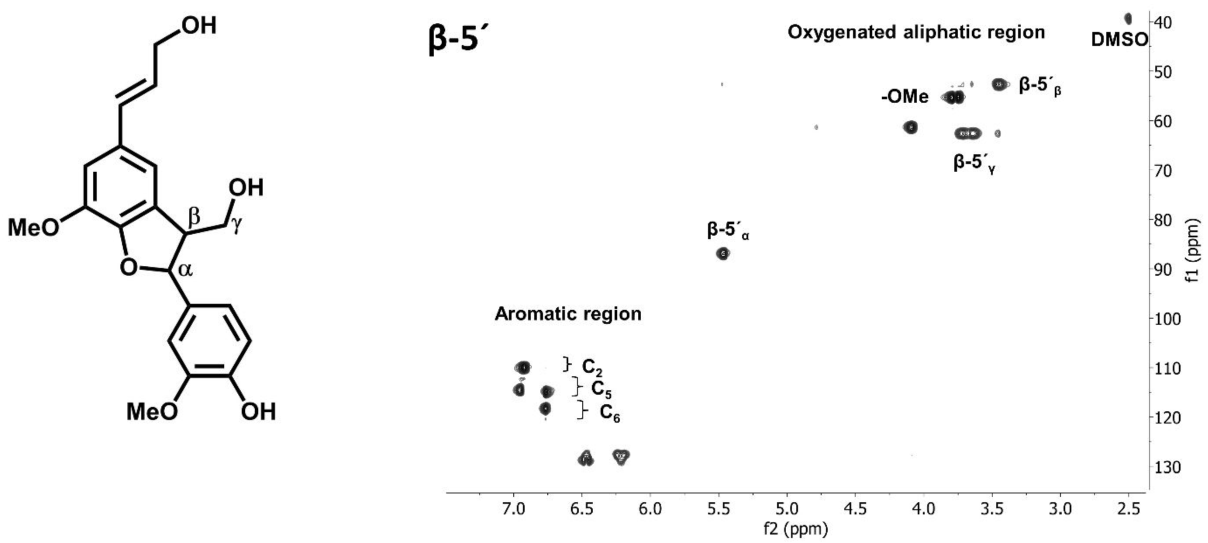

Figure S9. HSQC NMR spectrum of the β-5´ dimer, where f1 corresponds to the ^{13}C dimension and f2 to the ^{1}H dimension.

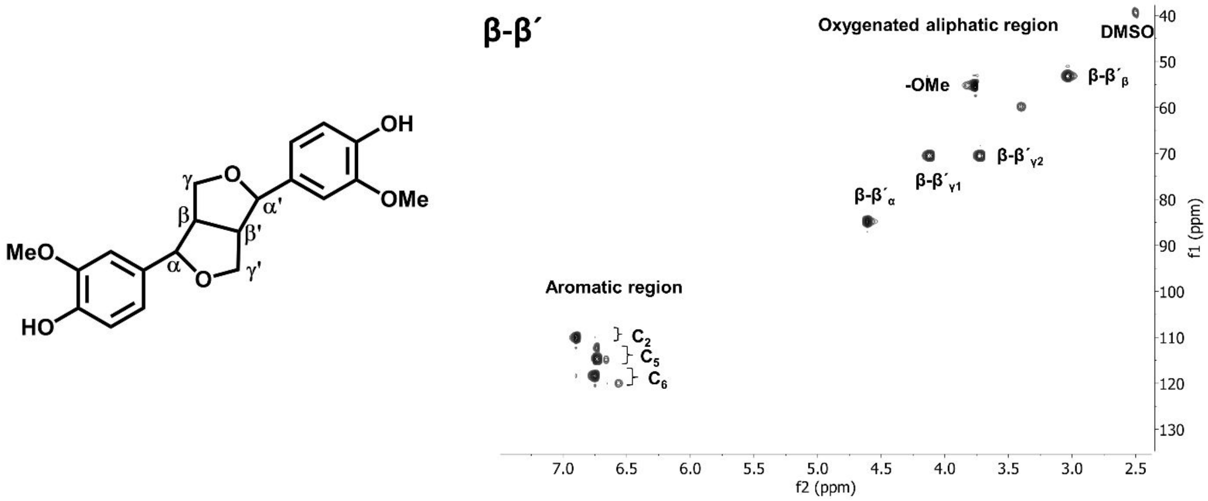

Figure S10. HSQC NMR spectrum of the β- β´ dimer, where f1 corresponds to the ^{13}C dimension and f2 to the ^{1}H dimension.

3.3 ^{31}P NMR of extracted lignin

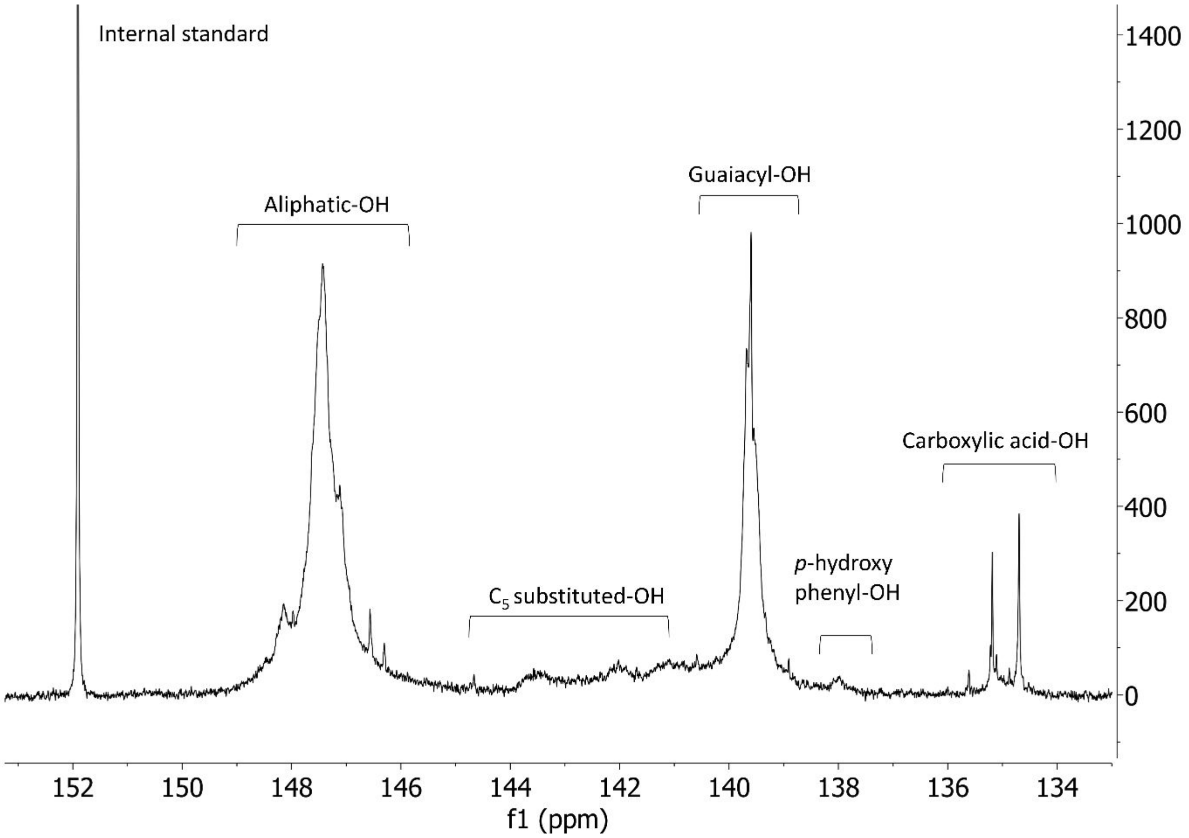

Figure S11. The ^{31}P NMR spectrum of the ethanol-soluble part of the extracted lignin sample. The quantified amount of aliphatic-OH was 3.1 mmol/g, C_5 substituted-OH 0.6 mmol/g, guaiacyl-OH 1.6 mmol/g, *p*-hydroxy phenyl-OH 0.06 and carboxylic acid-OH 0.3 mmol/g.

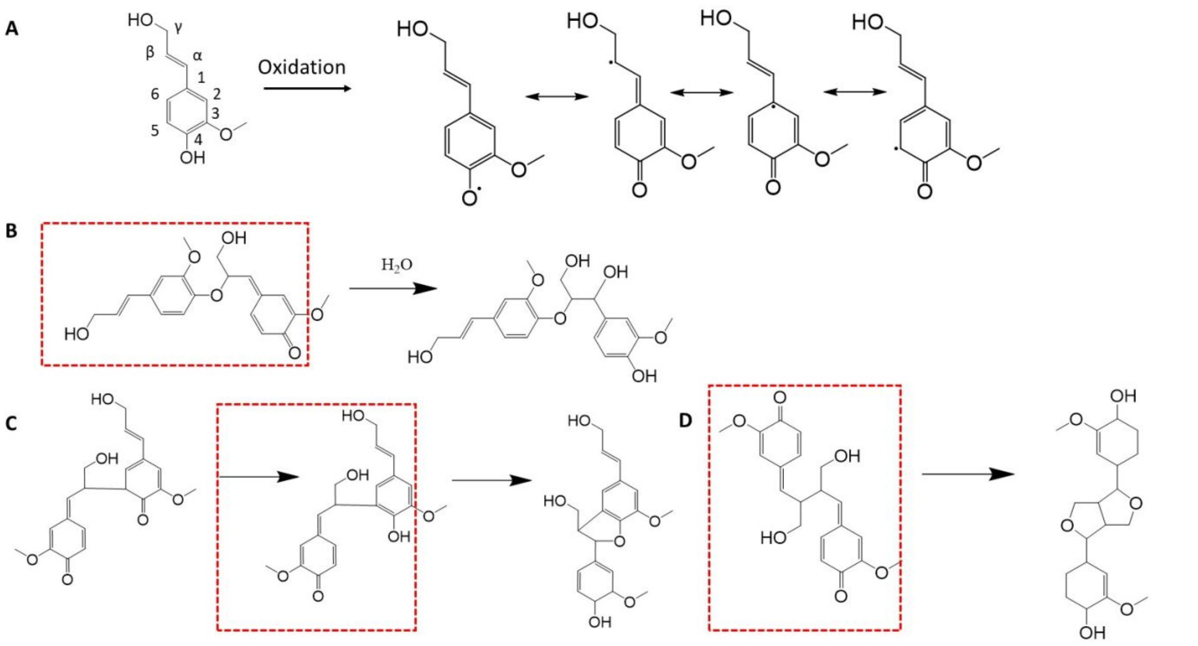

Figure S12. Key reactions in lignin polymerization. **A**: Oxidation of Monolignol to yield phenoxi-radical and its resonances. Shown are the important resonance forms that yield coupling. **B**: The β-O-4' linkage is formed by radical coupling of a phenoxy-radical to a β-radical, to form quinone methide (QM) intermediate, marked in red dotted rectangle. In this case, the addition of water as an external nucleophile, to QM is favorable to form the α-hydroxylated compound. **C**: The formation of Phenylcoumaran structure. This starts with the radical coupling to form β-5' linkage, rearomatization, and then an internal trapping reaction (intramolecular reaction) of QM by phenolic hydroxyl group to form the phenylcoumaran ring. **D**: Formation of Pinoresinol structure. This starts with radical coupling of two β radicals to form β-β' linkage, followed by internal trapping of the two QM intermediate moeieties by two adjacent pliphatic hydroxyls to form the pinoresinol ring structure.

References

1. Jawerth, M.; Lawoko, M.; Lundmark, S.; Perez-Berumen, C.; Johansson, M., Allylation of a lignin model phenol: a highly selective reaction under benign conditions towards a new thermoset resin platform. *RSC Adv.* **2016,** *6* (98), 96281-96288.

2. Ralph, S. A.; Ralph, J.; Landucci, L.; Landucci, L., NMR database of lignin and cell wall model compounds. *US Forest Prod. Lab., Madison, WI (http://ars. usda. gov/Services/docs. htm* **2004**.
